# Highlighting the Phototherapeutical Potential of Fungal Pigments in Various Fruiting Body Extracts with Informed Feature-Based Molecular Networking

**DOI:** 10.1007/s00248-023-02200-2

**Published:** 2023-03-22

**Authors:** Fabian Hammerle, Luis Quirós-Guerrero, Jean-Luc Wolfender, Ursula Peintner, Bianka Siewert

**Affiliations:** 1 Institute of Pharmacy/Pharmacognosy, Center for Molecular Biosciences Innsbruck (CMBI), University Innsbruck, Innrain 80/82, 6020 Innsbruck, Austria; 2 Phytochemistry and Bioactive Natural Products, School of Pharmaceutical Sciences, University of Geneva, CMU - Rue Michel-Servet 1, 1211 Geneva, Switzerland; 3 Institute of Pharmaceutical Sciences of Western Switzerland, University of Geneva, CMU, 1211 Geneva, Switzerland; 4 Department of Microbiology, University Innsbruck, Technikerstrasse 25d, 6020 Innsbruck, Austria

**Keywords:** Ascomycota, Basidiomycota, Fungal pigments, PDT, Photochemical defense, FBMN study, DMA‐assay

## Abstract

Fungal pigments are characterized by a diverse set of chemical backbones, some of which present photosensitizer-like structures. From the genus *Cortinarius*, for example, several biologically active photosensitizers have been identified leading to the hypothesis that photoactivity might be a more general phenomenon in the kingdom Fungi. This paper aims at testing the hypothesis. Forty-eight fruiting body-forming species producing pigments from all four major biosynthetic pathways (i.e., shikimate-chorismate, acetate-malonate, mevalonate, and nitrogen heterocycles) were selected and submitted to a workflow combining in vitro chemical and biological experiments with state-of-the-art metabolomics. Fungal extracts were profiled by high-resolution mass spectrometry and subsequently explored by spectral organization through feature-based molecular networking (FBMN), including advanced metabolite dereplication techniques. Additionally, the photochemical properties (i.e., light-dependent production of singlet oxygen), the phenolic content, and the (photo)cytotoxic activity of the extracts were studied. Different levels of photoactivity were found in species from all four metabolic groups, indicating that light-dependent effects are common among fungal pigments. In particular, extracts containing pigments from the acetate-malonate pathway, e.g., extracts from *Bulgaria inquinans*, *Daldinia concentrica*, and *Cortinarius* spp., were not only efficient producers of singlet oxygen but also exhibited photocytotoxicity against three different cancer cell lines. This study explores the distribution of photobiological traits in fruiting body forming fungi and highlights new sources for phototherapeutics.

**Supplementary Information:**

The online version contains supplementary material available at 10.1007/s00248-023-02200-2.

## Introduction

Of the estimated 2.2 to 3.8 million fungal species [1], only approx. 148,000 have been described [2], indicating enormous potential for phylogenetic and mycochemical discoveries. Numerous secondary metabolites have been isolated from fast-growing ascomycete fungi. Some significantly improved people’s daily lives, such as the antibiotic penicillin from *Penicillium rubens* [3] or the immunosuppressant cyclosporine from *Tolypocladium inflatum* [4]. In contrast, studies for the discovery of secondary metabolites from macrofungi have been neglected. Reasons behind are their complex genome structures and comparatively slow growth rates [5], as well as the intricate sourcing of sufficient amounts needed for isolation. As consequence, many mushrooms have been relegated from the world’s laboratories to scientists’ kitchens.

Nevertheless, basidiomycetes should not be reduced to their nutritional value only. They are known to produce compounds belonging to various biosynthetic classes (e.g., polyketides, terpenoids, peptides, and indole alkaloids) [6] and display remarkable bioactivities. For example, anticancer activities (e.g., ganoderic acid T from *Ganoderma lucidum* [7]) or hallucinogenic effects (e.g., psilocybin from *Psilocybe mexicana* [8]) are reported. Recent studies on the genus *Cortinarius* have even led to the discovery of an activity previously unknown for basidiomycetes: photoactivity [9, 10].

So far, it has been highlighted that fruiting body extracts from several *Cortinarius* species can inactivate microorganisms or kill cancer cells after irradiation with an appropriate light source and dose [9, 11, 12]. The dimeric anthraquinone 7,7′-biphyscion, active against A549 cancer cells in the nanomolar range after blue light irradiation, represents the most promising photosensitizer from basidiomycetes to date [12]. With its selective activity, 7,7′-biphyscion could become a lead structure for photodynamic therapy (PDT). In addition, evidence is accumulating that (i) ascomycetes produce photosensitizers too and that (ii) also these structures hold significant potential as hit structures of new phototherapeutic entities [13, 14] .

PDT is a unique treatment method for non-malignant and neoplastic diseases. Three different components, a light-sensitive compound, light, and oxygen—each harmless in itself—are combined into a bullet that efficiently and selectively targets cancer cells [15]. First, a light-absorbing molecule, a so-called photosensitizer (PS), is administered and ideally accumulates in the tumor tissue. Second, a fiber-optic device delivers light in a targeted manner to the respective area triggering a photochemical reaction, which can lead, e.g., to the conversion of harmless environmental triplet oxygen into cytotoxic singlet oxygen (type II PDT). Cancer eradication is finally mediated by direct cytotoxicity, vasculature damage, and the induction of an inflammatory reaction [16, 17]. Although the underlying scientific principle of PDT was discovered by Raab and Tappeiner as early as 1900 [18], it is still rarely used in modern medicine. Photosensitizers that have failed in clinical trials (e.g., lutrin, purlytin, or liposomal zinc phthalocyanine) and approved drugs with troublesome side effects (e.g., long-lasting skin photosensitivity induced by photofrin) [19] may be to blame. Thus, an overlooked source of natural photoactive products such as fungi could lead to the discovery of new photopharmaceuticals and eventually bring the long-awaited breakthrough [20].

Except for photoactive anthraquinone pigments from Cortinarii [21, 22], the occurrence of fungal photosensitizers in basidiomycetes is still a theoretical construct [23]. Thorough investigations on a metalevel are needed to acquire an overview of the putative trait distribution. A sensitive and reliable analytical technique capable of analyzing and processing large data sets from natural extracts is for example, the state-of-the-art mass spectrometry-based technique molecular networking (MN) [24] and its advancement feature-based molecular networking (FBMN) offering the possibility to visualize the complex metabolome of natural products as a molecular network comprising clusters of structurally related compounds [25]. Clusters associated with activity can be highlighted by adding biological data as a layer of information, which is termed bioactive natural products prioritization [26]. Subsequent annotation of these clusters is possible through mass spectral matching against experimental (e.g., Global Natural Products Social Molecular Networking (GNPS) library [27]), in silico generated databases (e.g., ISDB-DNP [28]), or via an elaborated approach anticipating unknown features from any identified compounds based on in silico generated metabolization [29]. In our recent study [30], the power of FBMN for annotating photoactive compounds was demonstrated by using a photophysical filter variable (i.e., the “VIS-Signal” variable).

The present study was intended to test the power of FBMN in the context of ecological research questions. In detail, we aimed at understanding and visualizing the distribution of a putative photochemical trait in macrofungi on a metalevel. Therefore, a total of 48 fungal species producing all known classes of fungal pigments were submitted to (i) a high-resolution mass spectrometry metabolite profiling and FBMN analysis to explore their chemical space, (ii) a photochemical assay to quantify their light-dependent singlet oxygen production, (iii) an assay to determine their phenolic content, and (iv) cell culture experiments to evaluate their (photo)cytotoxic activity.

## Material and Methods

### Chemicals and Instrumentation

All solvents for the extraction procedures were obtained from VWR International (Vienna, Austria). HPLC-grade solvents were purchased from Merck (Merck KGaA, Darmstadt, Germany). Petroleum ether was distilled prior to its use. The reagents sulforhodamine B (acid red 52, product number: A0600), 9,10-dimethylanthracene (product number: D0252), and rose bengal (acid red 94, product number: R0041) were purchased from TCI Deutschland GmbH (Eschborn, Germany). Folin-Ciocalteu reagent (catalog number: ICNA0219518690) and sodium carbonate for the Folin-Ciocalteu assay were purchased from VWR International (Vienna, Austria) and Merck (Merck KGaA, Darmstadt, Germany), respectively. Gallic acid and L-ascorbic acid were obtained from Merck (Merck KGaA, Darmstadt, Germany). A Sartorius arium® 611 UV purification system (Sartorius AG, Göttingen, Germany) was used to produce ultrapure water. Consumables, media, and supplements (e.g., flasks, DMEM, Opti-MEM^TM^, FCS, penicillin/streptomycin, trypsin, PCS, 96-well plates, etc.) needed for cell culture maintenance and the (photo)cytotoxicity assay were obtained from Thermo Fischer Scientific (Waltham, MA, USA) and SARSTEDT AG & Co. KG (Nümbrecht, Germany).

An Agilent E3611A DC Power Supply (Agilent Technologies, Inc., Santa Clara, USA) power adaptor in combination with two custom-made LED-panels (λ = 468 ± 27 nm (1.24 J cm^−2^ min^−1^) and λ = 519 ± 33 nm (1.34 J cm^−2^ min^−1^)) (University Leiden, published in Hopkins et al. [31]) were employed for the photophysical evaluation (DMA-assay). Absorption measurements were done with the plate reader Tecan Spark® 10 M (Tecan Group Ltd., Männedorf, Switzerland). A Mettler Toledo SevenMulti (Mettler-Toledo GmbH, Vienna, Austria) pH-meter was used to adjust pH-values. Sartorius Cubis®-series (Sartorius AG, Göttingen, Germany) and Mettler Toledo AB54 (Mettler-Toledo GmbH, Gießen, Germany) balances were used for weighing samples. A GFL 1042 (Gesellschaft für Labortechnik mbH, Burgwedel, Germany) heating bath was employed for the preparation of crude extracts by Soxhlet extraction. The ultrasonic baths Sonorex RK 52 (BANDELIN electronic GmbH & Co. KG, Berlin, Germany) and Sonorex RK 106 were used for ultra-sonic extraction. A Vortex-Genie 2 vortex mixer (Scientific Industries, Inc., Bohemia, NY, United States) was utilized to homogenize the samples. Vacuum rotary evaporation was carried out using a Heidolph Laborota 4000 efficient rotary evaporator (Heidolph Instruments GmbH & Co. KG, Schwabach, Germany) coupled to a vacuubrand PC 101 NT (VACUUBRAND GmbH & Co. KG, Wertheim, Germany) vacuum pump. For centrifugation, an Eppendorf 5804R centrifuge with a F-45–30-11–30 place fixed angle rotor (Eppendorf AG, Hamburg, Germany) was used. Pipettes and pipette tips were purchased from Eppendorf AG (Hamburg, Germany) or from STARLAB International GmbH (Hamburg, Germany). Further specific materials and equipment are listed in the respective sections.

### Fungal Biomaterial

A detailed list of all 48 investigated fungal species with information on their origin is provided in the supplementary information (SI Sect. 2, Table S1). The present study includes three species of Ascomycota, and 45 species of Basidiomycota (agarics, boletes, *Russula*, *Lactarius*, chanterelles, polypores, and corticoids). Authenticated voucher specimens are deposited in the Natural Sciences Collections of the Tiroler Landesmuseen (IBF) (Krajnc-Straße 1, 6060 Hall, Austria / http://www.tiroler-landesmuseen.at/page.cfm?vpath=index) or in the mycological collection of the Verein für Pilzkunde München e.V. (http://www.pilze-muenchen.de/index.html).

### Extract Preparation

Dried fungal biomaterial was ground to a fine powder using a Bosch TSM6A013B (Robert Bosch AG, Vienna, Austria) rotating blade coffee grinder. If large amounts of biomaterial were present, extracts were prepared by Soxhlet extraction, but if only small quantities were available, the extractions were performed by ultrasonication.

For Soxhlet extraction, powdered fruiting bodies (m ~ 2 g) were weighed into a filter holder and placed in a Soxhlet apparatus. The biomaterials were first defatted with petroleum ether (V = 450 mL, t = 8 h, T_water_ _bath_ = 70 °C). Thereafter, they were dried overnight and extracted with methanol the following day (V = 450 mL, t = 8 h, T_water_ _bath_ = 80 °C). Solvents were removed using vacuum rotary evaporation at 40 °C and the obtained extracts were dried under an airstream.

*Cytidia salicina*, *Ganoderma lucidum*, *Hypomyces lactifluorum*, and the three examined *Tricholoma* species (*T. bufonium*, *T. equestre*, and *T. sulphureum*) were subjected to ultra-sonic extraction. The *C. salicina* extract was obtained using the following protocol: the ground sample (m = 7.5 mg) was extracted with methanol (V = 10 mL) via ultra-sonication (t = 5 min), followed by centrifugation (t = 5 min, relative centrifugal force (RCF) = 2000 × g) and filtration of the supernatant through cotton wool. The procedure was repeated twice, whereby the filtered supernatants were combined and dried under an air stream. All steps were done under the exclusion of direct light. The ground fruiting bodies of *G. lucidum* and the pulverized *H. lactifluorum* material (m ~ 7.0 g) were sequentially ultrasonicated with petroleum ether (V_1_ = 300 mL, V_2&3_ = 150 mL, t_extraction step_ = 10 min, *n* = 3) and methanol (V_1_ = 300 mL, V_2&3_ = 150 mL, t_extraction step_ = 10 min, *n* = 3). The respective extracts were filtered, combined, concentrated in vacuo, and dried under an air stream.

The fruiting bodies of *T. bufonium* and *T. sulphureum* were extracted as follows: powdered biomaterial (m ~ 1 g) was extracted with petroleum ether (V = 5 mL) for 5 min via ultra-sonication, centrifuged (t = 5 min, RCF = 2000 × g), and the supernatant filtered through cotton wool. The procedure was conducted four times, filtered supernatants were collected, and the combined extract was dried under an air stream. The biomaterial was dried overnight and then extracted with methanol as described above (V_methanol_ = 5 mL, t_ultra-sonic extraction_ = 5 min, t_centrifugation_ = 5 min).

The *T. equestre* extract was prepared in a larger scale (m_biomaterial_ = 59 g) as it was also used for another study. The ground fruiting bodies were defatted with petroleum ether (ultra-sonic extraction: V = 500 mL, t = 10 min, *n* = 2; maceration: V = 500 mL, t = 15 h), followed by extraction with dichloromethane (ultra-sonic extraction: V = 500 mL, t = 10 min, *n* = 3; maceration: V = 500 mL, t = 89 h). All extraction yields are given in the supplementary in tabular form (SI Sect. 3, Table S2).

#### DMA Assay

The photophysical characterization of fungal extracts using the 9,10-dimethylanthracene (DMA) assay was done as published previously [9]. The extracts were dissolved in DMSO (c = 10 mg/mL) and tested for their blue and green light-induced photoactivity (i.e., generation of singlet oxygen (^1^O_2_)). The yellow pigment berberine (c = 1.0 mg/mL, 2.7 mM, V = 10 µL, DMSO, λ_max_ = 420 nm, Φ_Δ, DCM_ = 0.25, Φ_Δ, EtOH_ = 0.04) [32, 33] and the pink pigment rose bengal (c = 0.1 mg/mL, 0.10 mM, V = 10 µL, DMSO, λ_max_ = 555 nm, Φ_Δ, EtOH_ = 0.86) [32] were used as positive controls for the blue (λ = 468 ± 27 nm) and green light irradiation group (λ = 519 ± 33 nm), respectively. False positives were excluded by using the ^1^O_2_-quenching compound ascorbic acid. DMSO was used as negative control. Optical densities at the wavelengths 377 nm, 468 nm, and 519 nm were measured with a plate reader. The irradiation was conducted as follows: blue light—four irradiation cycles at t = 5 min each (H_total_ = 24.8 J cm^−2^); green light—four irradiation cycles at t = 4.6 min each (H_total_ = 24.7 J cm^−2^). All measurements were carried out as technical duplicates. The light-dependent ^1^O_2_ generation of an extract was calculated relative to the respective positive control as published elsewhere [9].

The petroleum ether extracts of *Albatrellus cristatus*, *Amanita muscaria*, *Cortinarius cinnabarinus*, *C. olivaceofuscus*, *C. purpureus*, *C. semisanguineus*, *C. uliginosus*, *Gymnopilus penetrans*, and *Inonotus hispidus*, as well as the methanol extract of *Cytidia salicina* were tested at a concentration of c = 1 mg/mL, as sample consumption had to be minimized.

#### Determination of Polyphenolic Content (Folin-Ciocalteu Assay)

The polyphenolic content was measured according to a modified Folin-Ciocalteu assay protocol by Gautam et al. [34]. Reagent amounts were adjusted so that the assay could be performed in a 96-well plate format for high-throughput screening of the fungal extracts. The Folin-Ciocalteu’s reagent (V = 110 µL, c = 2 mol/L) was mixed (orbital shaking, t = 10 s, frequency = 510 rpm, amplitude = 1 mm) with the fungal extract solution (V = 45 µL, c = 1 mg/mL in HPLC-grade methanol). After an incubation period of t = 3 min, saturated sodium carbonate solution (V = 45 µL) was added. Then, the mixture was homogenized (shaking parameters as above) and incubated for another 2 h. Thereafter, the optical density at λ = 725 nm was measured. The polyphenolic content was calculated using a calibration curve (*r*^2^ = 0.9904) established with gallic acid as the reference compound and expressed in µg/mL gallic acid equivalents. Mean values were determined from three technical repetitions.

#### (Photo)cytotoxicity Evaluation

Cells of the adherent cancer cell lines A549 (non-small cell lung cancer, ATCC, Sigma-Aldrich), AGS (stomach cancer, CLS, Eppelheim), and T24 (urinary bladder carcinoma, CLS, Eppelheim) were cultivated in 75 cm^2^ Nunc EasYFlasks (product number: 51985042, 75 cm^2^) with Gibco™ MEM-medium (product number: 42360081) supplemented with fetal calf serum (FCS, 10% v/v) and penicillin/streptomycin (P/S, 1% v/v). The cells were trypsinized every other day at approximately 80% confluency and used for 8–12 weeks. Cell cultures were thawed/frozen according to standard protocols. The (photo)cytotoxic activity of fungal extracts was determined as published elsewhere [9, 31].

The assay was conducted as follows: cells (AGS: 2500 cells/well, T24 & A549: 2000 cells/well) were seeded in 96-well plates in Gibco™ Opti-MEM (OMEM, product number: 11058021) containing FCS (2.4% v/v) and P/S (1% v/v) at 37 °C in 5% CO_2_ atmosphere. For the initial screening, fungal extracts were dissolved in DMSO (c_stock solution_ = 10 mg/mL) and further diluted with supplemented OMEM. The cancer cells were treated 24 h after the seeding step with three working solutions per extract (final concentrations: 50, 25, and 5 µg/mL), followed by an incubation period. As a negative control, the cells were treated with supplemented OMEM only. After 24 h, the working solutions or the old medium were aspirated and replaced with fresh, supplemented OMEM. To differentiate between light-dependent and light-independent/dark cytotoxicity, two identically treated plates were tested. One was irradiated with blue light for 7.5 min (λ = 468 nm ± 27 nm, H = 9.3 J cm^−2^). The other one was left in the dark. After an additional incubation period of 48 h, the cells were fixed by gently adding cold (T = 8 °C) trichloroacetic acid (10% w/v in water, V = 100 µL/well) and stored in a refrigerator at T = 8 °C for at least 24 h. The fixed cell-monolayers were washed with slow running deionized tap-water and stained with sulforhodamine B (SRB) (acid red 52, 0.4% w/v SRB in 1% v/v acetic acid, V = 100 µL/well) for 30 min. Thereafter, the plates were washed five times with 1% v/v acetic acid and dried at room temperature. Then, the dried dye was dissolved in tris(hydroxymethyl)aminomethane-solution (TRIS, c = 10 mM, V = 100 µL/well, ultrapure water) and incubated for at least 20 min. Absorbance was measured at λ = 540 nm with a plate reader. Data analysis was performed using Excel 365 (Microsoft Corporation, Redmond, USA), identifying the interval of extract concentration (i.e., +  + , 0.01–5 µg/mL; + , 5–25 µg/mL; o, 25–50 µg/mL; -, > 50 µg/mL) at which a 50% reduction in cancer cell population was observed. Measurements were done as technical triplicates.

The extracts of *Bulgaria inquinans* and *Daldinia concentrica* were selected for further testing. Biological triplicate measurements were performed with six working solutions (final concentrations: c = 55.0, 27.5, 11.0, 5.50, 2.75, and 0.55 µg/mL) as described above. EC_50_ values, including their confidence intervals (95%), were calculated with GraphPad Prism 5 employing the relative Hill-Slope equation (“log(inhibitor) vs. normalized response – variable slope”). The ratio of cells selectively killed by the combination of extract and blue light irradiation to the cells killed in the absence of light (i.e., extract dark-cytotoxicity), is represented by the photoindex (P.I.) and calculated using following equation: P.I. = EC_50|dark_ / EC_50|irradiated_. Micrographs of the treated cancer cells are given in the electronic supplementary information (SI Sect. 6).

#### UHPLC-MS/MS Analysis and Data Treatment

Prior to the UHPLC-HRMS/MS measurement, the extracts were subjected to a solid-phase extraction pre-treatment protocol using Strata® C18-E (55 µm, 70 Å, 50 mg/1 mL) SPE cartridges from Phenomenex® (Phenomenex Inc., Aschaffenburg, Germany). First, the cartridges were activated with HPLC-grade methanol (V > 3 mL) and then equilibrated with at least 3 mL of methanol/ultrapure water (9:1 v/v). The methanolic extracts (m ~ 10 mg) were dissolved in methanol/ultrapure water (9:1 v/v, V = 10 mL) and loaded onto the equilibrated column. Elution was performed with 5 mL of methanol 90%, followed by lyophilization.

UHPLC-HRMS/MS analysis was done on a Waters Acquity UPLC system hyphenated to a Q-Exactive Focus mass spectrometer (Thermo Scientific, Bremen, Germany), using a heated electrospray ionization (HESI-II) source and a CAD detector (Thermo Scientific, Bremen, Germany). The instrument was controlled using Thermo Scientific Xcalibur 3.1 software. A Waters Acquity BEH C18 50 × 2.1 mm, 1.7 µm column was used as stationary phase. Water with 0.1% formic acid (A) and acetonitrile with 0.1% formic acid (B) were chosen as mobile phases. The analysis was performed using a linear gradient of 5–100% B over 7 min and an isocratic step at 100% B for 1 min. Flow rate and injection volume were set to 600 µL/min and 2 µL, respectively. ESI parameters were as follows: source voltage, 3.5 kV (positive and negative); sheath gas flow rate (N_2_), 55 units; auxiliary gas flow rate, 15 units; spare gas flow rate, 3.0; capillary temperature, 350.00 °C, S-Lens RF Level, 45. The mass analyzer was calibrated using a mixture of caffeine, methionine-arginine-phenylalanine-alanine-acetate (MRFA), sodium dodecyl sulfate, sodium taurocholate, an Ultramark 1621 in an acetonitrile/methanol/water solution containing 1% formic acid (Pierce™ LTQ ESI Positive Ion Calibration Solution, Thermo Scientifuc, Bremen, Germany) by direct injection. The data-dependent MS/MS events were performed on the three most intense ions detected in full scan MS. The MS/MS isolation window width was 1 Da, and the stepped normalized collision energy (NCE) was set to 15, 30, and 45 units. In data-dependent MS/MS experiments, full scans were acquired at a resolution of 35 000 FWHM (at *m/z* 200) and MS/MS scans at 17,500 FWHM both with an automatically determined maximum injection time. After being acquired in a MS/MS scan, parent ions were placed in a dynamic exclusion list for 2.0 s.

The data obtained from the UHPLC-MS/MS measurement were converted from RAW (Thermo) standard data format to mzXML format using the MS Convert software [35]. The files were treated in MZmine v. 2.53 [36]. The parameters used were as follows: for mass detection, a MS1 noise level of 1.0E5 for positive (1.0E4 for negative), and 0.0E0 for MS2 level (both modes) were chosen. The ADAP chromatogram builder was used and set to a minimum group size of scans of 5, minimum group intensity threshold to 1.0E5 for positive (1.0E4 for negative), and the minimum highest intensity to 1.0E5 for positive (1.0E4 for negative). The *m/z* tolerance was set to 0.005 m*/z* or 12.0 ppm for both ionization modes. Chromatogram deconvolution was performed using the ADAP algorithm (wavelets). The intensity window S/N was used as S/N estimator with a signal to noise ratio set to 10, a minimum feature height of 1.0E5, a coefficient area threshold of 110, peak duration ranges from 0.01 to 1.0 min, and the RT wavelet range from 0.01 to 0.08 min. The *m/z* and RT ranges for MS2 scans pairing were set to 0.025 and 0.15, respectively. Using the Peak filter, all peaks not bearing a MS2 spectrum associated were removed. Isotopes were detected using the isotopes peaks grouper with an *m/z* tolerance of 5.0 ppm, a RT tolerance of 0.05 min (absolute), the maximum charge set to 1 and the representative isotope used was the most intense. Alignment was performed using the Join Aligner algorithm. A *m/z* tolerance of 0.005 or 8 ppm and a RT tolerance of 0.05 min was used. Additionally, the Compare Spectral Similarity option was used. The spectral tolerance was set to 0.005 m*/z* or 8 ppm. The MS level 2 and the algorithm used was weighted dot-product cosine, with the default parameters. The resulting files were filtered using the peak-list rows filter option to keep features between 0.40 and 7 min for positive mode and between 0.25 and 7.40 for negative mode. A custom-made database of fungal pigments (*n* = 684) based on several review articles [37–42] was used for dereplication, whereby a *m/z* tolerance of 8.0 ppm was applied.

The level of confidence achieved in the annotation of the features was indicated by the four levels of accuracy defined by the Metabolomics Standard Initiative [43].

The raw data are available in the following MassIVE repository (https://massive.ucsd.edu/ProteoSAFe/static/massive.jsp): MSV000088756 [10.25345/C5DC69].

#### Molecular Network Generation and Taxonomically Informed Metabolite Annotation

A molecular network was created with the FBMN workflow [25] on GNPS (https://gnps.ucsd.edu [27]). The results were exported to GNPS for FBMN analysis from MZmine. The precursor ion mass tolerance was set to 0.02 Da and the MS/MS fragment ion tolerance to 0.02 Da. A molecular network was then created where edges were filtered to have a cosine score above 0.7 and more than 6 matched peaks. Furthermore, edges between two nodes were kept in the network only if each of the nodes appeared in the respective top 10 most similar nodes of the other. Finally, the maximum size of a molecular family was set to 100, and the lowest scoring edges were removed from molecular families until the molecular family size was below this threshold. The spectra in the network were then searched against GNPS spectral libraries [27, 44]. All matches kept between network spectra and library spectra were required to have a score above 0.7 and at least 6 matched peaks. The job can be found in the following link: https://gnps.ucsd.edu/ProteoSAFe/status.jsp?task=1b8b31b8ff424f3ca1ceabd90b9750f7.

The results from the molecular network were subjected to dereplication against an in silico database of combined information from the Lotus [45] and Dictionary of Natural Products (DNP, https://dnp.chemnetbase.com/faces/chemical/ChemicalSearch.xhtml) databases. After spectral matching, the putative identifications were re-ranked considering the taxonomical information from the samples [46]. The in silico database contained the chemical ontology for all the entries, and the script performed the calculation to obtain the consensus by cluster.

## Results and Discussion

### Study Overview

**Fig. 1:**
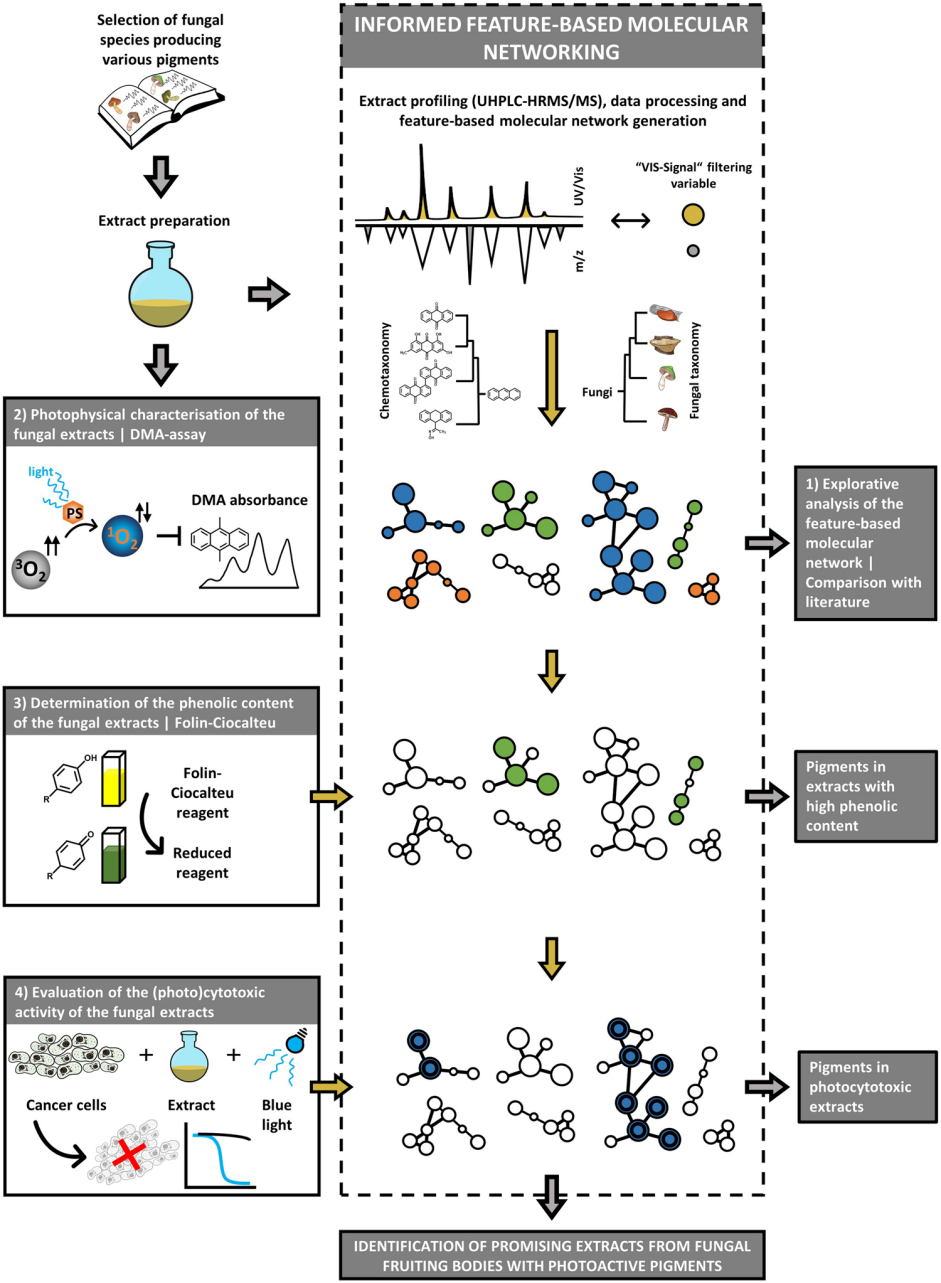
Graphical representation of the key points and structure of the presented study. Starting from an informed selection of 48 fruiting body-forming fungi, extracts were prepared and submitted to an extract profiling utilizing feature-based molecular networking (FBMN) analysis. In the first analytical step (1), the chemical space was explored and the obtained annotations compared to the literature. In the second step (2), a high-throughput assay was conducted testing the extracts’ abilities to generate singlet oxygen. In step (3), the phenolic content of the extracts was studied and the respective pigments highlighted in the FBMN. Finally, in step (4), the (photo)cytotoxic activity was determined and the results integrated into the FBMN, leading to the identification of promising species and fungal PSs

The present study was designed to test on a metalevel the hypothesis that a photochemical trait is prevalent in fruiting body-forming fungal species. Several assays were conducted (Fig. 1), and the results were interwoven: First, the biomaterial was selected based on the classification of fungal pigments used in a review article by Gill and Steglich [37] focusing on their biosynthetic origin. Forty-eight different species were selected to cover most of the described pigment types. Second, dried and ground fruiting bodies were extracted and subjected to untargeted UHPLC-HRMS/MS metabolite profiling together with previously isolated fungal photosensitizers [10, 12]. The obtained data was used to generate a FBMN [25]. To explore the secondary metabolites present in the fungal extracts, in silico metabolite annotation was performed incorporating (chemo)taxonomic information [46, 47] (Fig. 1 (1)). For rapid and cell-free determination of the green and blue light-dependent singlet oxygen production (i.e., photoactivity), the extracts were tested using the DMA assay (Fig. 1 (2)). The Folin-Ciocalteu assay was employed to quantify the phenolic content of the extracts (Fig. 1 (3)). Finally, the extracts were subjected to a (photo)cytotoxicity assay using three different cancer cell lines (Fig. 1 (4)) to evaluate the general activity of the extracts in a biological setting and to validate the findings obtained from the photochemical experiments (i.e., the DMA assay).

#### FBMN

The selected fungal species used in this study contain pigments derived from all major biosynthetic pathways (i.e., based on the review article of Gill and Steglich [37]: shikimate-chorismate pathway (SCP), acetate-malonate pathway (AMP), mevalonate pathway (MP), and nitrogen heterocycles (NH)). To ensure that the pigments described were present in the extracts produced, all methanolic extracts were analyzed by a generic untargeted metabolite profiling by UHPLC-HRMS/MS in positive ionization mode. The data were organized using FBMN [25] to group features by spectral similarity, highlight common compounds between extracts, and perform dereplication.

A molecular network was generated using the Global Natural Products Social Molecular Networking (GNPS) platform [27]. The network comprised 7348 individual nodes and 11,882 edges. Thereafter, experimental MS^2^ data (i.e., the GNPS spectral library [27]) as well as in silico fragmentation spectra of a comprehensive set of natural products (ISDB-DNP [28]) were used to annotate the MS^1^ and MS^2^ spectra. A script for taxonomically informed metabolite annotation [46] was employed to re-rank the candidate annotations and automatically assign the NPClassifier chemical taxonomy [47] to the dataset. The annotation against the GNPS spectral library yielded 539 putative structural suggestions; thus, a match was found for 7% of all features. A higher annotation hit rate (i.e., 14%) was found by matching the data with in silico spectra (ISDB-DNP). Overall, however, only a small number of features were identified. Quality issues with the obtained raw data can be excluded as cause due to the following facts: (i) The acquisition parameters were tuned to maximize the number of high-quality fragmentation spectra [48], ensuring a good match with the in silico databases. (ii) An exclusion list was created from the solvent to avoid the generation of unwanted MS^2^ spectra. (iii) The FBMN acquired in negative ionization mode was additionally explored but no other relevant information was found.

Another reason for the low annotation rate might be the libraries used. The public GNPS spectral library contains user-submitted MS^2^ spectra and covers only 2.5% of all known natural products [49]. The in silico database, based on the Lotus database and the Dictionary of Natural Products, has more than 800,000 structure-organism spectra, but only 74,126 (approx. 9.3%) account for the kingdom Fungi and 20,607 (approx. 2.5%) for the division Basidiomycota. Nevertheless, as traditional approaches are gradually overtaken by untargeted metabolomics [5], a significant improvement can be anticipated if we all start filling public libraries such as the GNPS.

For annotation analysis on the meta-level of substance classes and biosynthetic pathways, the NPClassifier taxonomy [47] and the in silico based annotation results were utilized. A consensus was determined for each level by considering all the putative identities and the topology (i.e., the clusters) of the network. On the level “pathway” (i.e., biosynthetic pathway of the respective compound), the annotation process identified 530 features (f) as “terpenoids,” 198 f as “fatty acids,” 137 f as “polyketides,” 79 f as “alkaloids,” 71 f as “shikimates and phenylpropanoids,” and 11 f as “amino acids and peptides.” The remaining 23 annotated features were distributed among various groups with group sizes of less than eight features each. The investigation on the “class” level revealed the majority of identified features as “lanostane, tirucallane, and euphane triterpenoids” (161 f), followed by “ergostane steroids” (140 f), “anthraquinones and anthrones” (82 f), “unsaturated fatty acids” (69 f), “wax monoesters” (42 f), and “prenyl quinone meroterpenoids” (31 f).

The fatty acids group and their derivatives (from the acetate-malonate pathway (AMP)), i.e., unsaturated (69 f), wax monoesters (42 f), and fatty alcohols (20 f), was the largest group of polyketide compounds with more than 130 features. Besides fatty acids, heptaketides such as “naphthoquinones” (8 f) and octaketides such as “anthraquinones and anthrones” (82 f) were found. Originating from the shikimate-chorismate pathway (SCP), several features were identified as “p-terphenyls” (19 f) or “kavalactones and derivatives” (18 f). In addition, a small number of features were putatively annotated as alkaloids (i.e., nitrogen heterocycles (NH)), e.g., as “pyridine alkaloids” (12 f), “cytochalasan alkaloids” (11 f), “piperidine alkaloids” (10 f), and “simple indole alkaloids” (8 f).

To spot features capable of absorbing light in the visible spectral range, i.e., colored compounds or pigments, the filtering variable “VIS-Signal” was added as an informational layer [30]. First, the peaks of all chromatograms (λ_det_ = 468 nm) were analyzed to generate a set of peak lists containing the start and end time point of each peak. In parallel, the corresponding relevant extract was identified for each feature, i.e., the one in which the feature was most dominant. Subsequently, the retention time of each feature was compared to the peak list of the relevant extract. In case of a match, the feature was assigned with positive “VIS-Signal” and thus identified as a potential pigment. On the “pathway” level, the largest number of “VIS-Signal”-positive features were found for “polyketides” (31 f), followed by “terpenoids” (27 f), “shikimates and phenylpropanoids” (15 f), “fatty acids” (15 f), and “alkaloids” (9 f). Looking at the "class" level, most of the features exhibiting a “VIS-Signal” were distributed among “ergostane steroids” (16 f), “anthraquinones and anthrones” (16 f), and “p-terphenyls” (14 f). However, “fatty acids” and “ergostane steroids” are not colored compounds, so their positive “VIS-Signal” indicates the coelution of several compounds. This corresponds well with our mycochemical experience gained from isolating pigments. Anthraquinones, for example, are eluted frequently with other apolar components such as fatty acids. The effect could be partially reduced via defatting the fractions in a first step (data not shown).

**Fig. 2:**
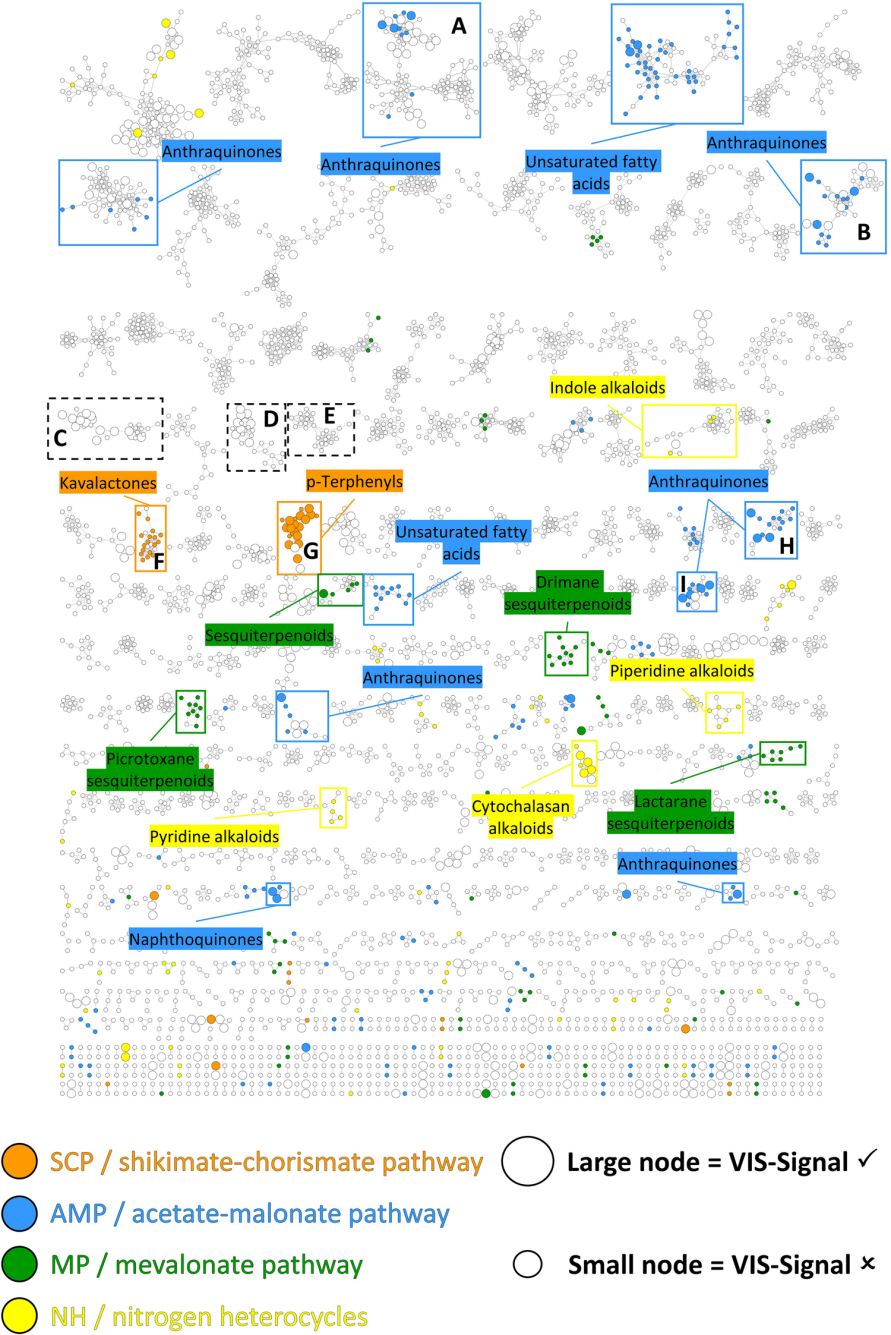
Feature-based molecular network (mass spectra acquired in positive ionization mode) visualized using Cytoscape. Features that were putatively annotated as compounds belonging to one of the four biosynthetic pathways (i.e., SCP, AMP, MP, and NH) were highlighted. In addition, features absorbing light in the visible spectral range (i.e., VIS-Signal present, λ_det_ = 468 nm) are depicted as large nodes, whereas a smaller node indicates the absence of absorbance. Clusters subjected to further annotation or discussed in the manuscript are marked A-I. For the sake of simplicity, the large number of individual nodes is not shown in the figure

Figure 2 depicts the FBMN with an informational layer comprising the NPClassifier “class” level and the “VIS-Signal” variable, thus comprehensively summarizing the findings discussed above. Compounds derived from all four biosynthetic pathways (i.e., SCP, AMP, MP, and NH) were detected and putatively annotated. The filtering variable “VIS-Signal” identified several features as potential pigments, which presumably belong to pigment classes described by Gill and Steglich (e.g., “anthraquinones and anthrones” and “p-terphenyls”). This indicated, in a first approximation, that the analyzed extracts contained the anticipated metabolites.

Nevertheless, the total number of “VIS-Signal”-positive features (independent from the annotation status) was, with 634 out of 7348 features (8.6%), relatively low. This might be due to several reasons originating from the Beer–Lambert law. One of them could be a (i) low pigment concentration in the extracts and, therefore, a low-intensity signal generated by the DAD detector. Pigments often occur in a small overall amount in the fruiting body and sometimes only in specific fruiting body tissues (e.g., in the cap of the fly agaric). The separation of the fungal tissues, however, was unfeasible in this study, as many fruiting bodies were sourced from different herbaria as dried material. Another reason for the low "VIS-signal" might be (ii) the selected single wavelength of λ = 468 nm. To obtain optimal results for a complete pigment assignment, the “VIS-Signal” should be generated from a full PDA chromatogram (λ = 400–800 nm). Unfortunately, the detector used could only detect in the visible range of λ = 400–500 nm, which limited the possibilities for this investigation. In addition, (iii) the major difference in sensitivity of the DAD and the MS detector must be considered. Sophisticated splitting approaches, however, will solve this small drawback. In sum, the “VIS-Signal” provided a sufficient mean to spot the main pigments in the FBMN, while traces of pigments were not yet directly highlighted. Nevertheless, due to the cluster generation, pigment clusters could be easily spotted (Fig. 2) and thus streamlined the annotation process.

Annotation beyond the “class” level, i.e., the identification of the molecular structure, is described in the respective following sections (for additional information, refer to SI Sect. 4). Special focus was directed towards clusters exhibiting features with a positive “VIS-Signal” variable (i.e., potential pigments) (clusters A, B, F–I; Fig. 2; SI Figure S1). In general, structures were selected based on the output of the taxonomically informed screening of experimental MS^2^ spectra against an in silico database of natural products (ISDB-DNP). In addition, or if the annotation based on MS^2^ did not yield a result, the corresponding accurate mass of features was matched against an in-house MS^1^ library of fungal pigments. The last step was used only in clusters exhibiting a certain level of structural homogeneity, i.e., with a “class” consensus.

### Photophysical Characterization

**Fig. 3:**
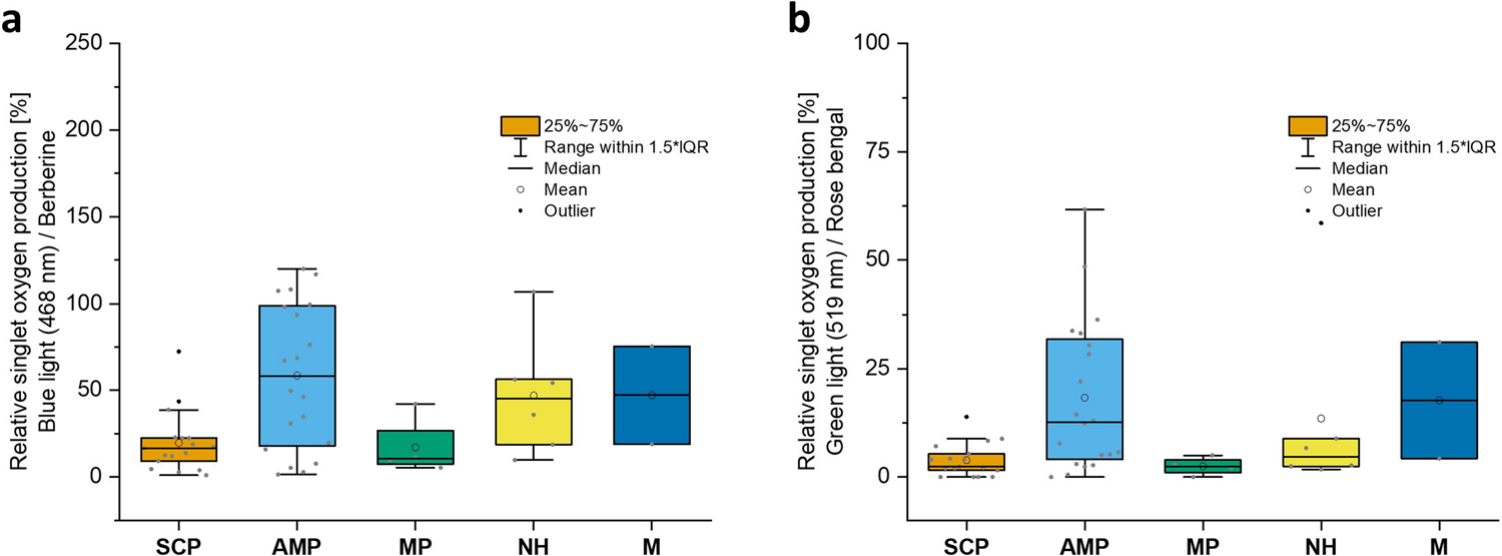
Boxplots, representing the results of the photophysical investigation (i.e., relative singlet oxygen production) of the polar methanolic extracts using the DMA-assay (**a** blue light irradiation, **b** green light irradiation). SCP = shikimate-chorismate pathway; AMP = aetate-malonate pathway; MP = mevalonate pathway; NH = nitrogen heterocycles; M = miscellaneous

The investigation of the fungal extracts’ ability to produce singlet oxygen (^1^O_2_) was conducted with a low-cost medium-throughput assay [9] employing DMA as chemical probe [50, 51] and a blue or green irradiation source. Descriptive statistics were used for data analysis of all extracts on the “pathway” level (Fig. 3). The full results of the photophysical characterization are provided in Table 1.

**Table 1:** Results of the photophysical investigation of the tested fungi sorted by biosynthetic pathway of the species' main pigments. The singlet oxygen production of the petroleum ether (PE) and methanol (MeOH) extracts solved in DMSO are given relative to the respective reference compounds (λ = 468 ± 27 nm, H = 24.8 J cm^−2^: relative to berberine, λ = 519 ± 33 nm, H = 24.7 J cm^−2^: rel. to rose bengal) (n.d., not determined due to insufficient sample quantity). Bold are highlighted all fungi with a photoactivity from > 50% in at least one setting

		Relative singlet oxygen production			
Pathway group	Fungus	PE 468 nm	MeOH 468 nm	PE 519 nm	MeOH 519 nm
Pigments from the Shikimate-Chorismate Pathway (SCP)	*Albatrellus cristatus*	8.2%	2.8%	3.5%	2.4%
	*Amanita muscaria*	7.4%	9.2%	1.7%	1.9%
	*Boletus erythropus*	11.8%	12.6%	2.4%	2.2%
	*Fomes fomentarius*	25.2%	38.7%	8.5%	13.9%
	*Gymnopilus penetrans*	6.3%	43.6%	3.9%	7.1%
	*Hygrocybe conica*	20.9%	3.9%	2.4%	0.0%
	*Hygrocybe punicea*	21.2%	4.5%	2.4%	0.0%
	*Inonotus hispidus*	7.0%	1.0%	3.2%	1.6%
	***Paxillus atrotomentosus***	**32.5%**	**72.4%**	**3.0%**	**0.0%**
	*Paxillus involutus*	19.8%	18.8%	2.9%	4.2%
	*Phanerochaete sanguinea*	21.0%	22.5%	8.1%	8.4%
	*Pholiota flammans*	31.0%	16.5%	6.8%	8.8%
	*Suillus bovinus*	36.5%	17.3%	8.5%	4.0%
	***Suillus collinitus***	**55.7%**	**22.4%**	**12.4%**	**5.3%**
	*Suillus grevillei*	22.5%	22.8%	3.0%	4.0%
	*Suillus luteus*	27.1%	13.8%	5.9%	2.0%
	*Xerocomus rubellus*	13.3%	12.1%	2.4%	0.0%
Pigments from the acetate-malonate pathway (AMP)	***Bulgaria inquinans***	**75.0%**	**68.7%**	**21.9%**	**28.3%**
	***Cortinarius cinnabarinus***	**296.1%**	**98.2%**	**14.0%**	**48.5%**
	***Cortinarius cinnamomeoluteus***	**102.7%**	**107.4%**	**55.2%**	**33.8%**
	*Cortinarius magicus*	19.1%	7.7%	5.0%	2.7%
	***Cortinarius malicorius***	**116.6%**	**99.5%**	**49.0%**	**12.9%**
	***Cortinarius purpureus***	**43.4%**	**67.1%**	**9.5%**	**22.0%**
	***Cortinarius rufo-olivaceus***	**65.1%**	**46.2%**	**7.7%**	**12.3%**
	***Cortinarius semisanguineus***	**80.8%**	**93.6%**	**16.3%**	**33.2%**
	***Cortinarius trivialis***	8.2%	5.2%	3.8%	3.0%
	***Cortinarius uliginosus***	**101.2%**	**108.2%**	**3.8%**	**30.4%**
	*Cortinarius venetus*	7.0%	19.7%	2.1%	0.0%
	***Cortinarius xanthophyllus***	**104.4%**	**49.6%**	**11.6%**	**14.4%**
	***Cortinarius olivaceofuscus***	**101.8%**	**116.7%**	**2.1%**	**36.4%**
	*Cytidia salicina*	n.d	16.0%	n.d	5.7%
	***Daldinia concentrica***	**210.6%**	**119.9%**	**79.5%**	**61.7%**
	***Hypomyces lactifluorum***	5.3%	1.5%	3.4%	0.5%
	***Tricholoma bufonium***	27.5%	34.8%	3.1%	5.1%
	***Tricholoma equestre***	**36.4%**	**76.3%**	**3.3%**	**7.7%**
	***Tricholoma sulphureum***	16.8%	30.9%	2.4%	5.2%
Pigments from the mevalonate pathway (MP)	*Cantharellus lutescens*	2.1%	5.4%	4.7%	2.9%
	*Craterellus cornucopioides*	34.6%	9.5%	1.3%	2.1%
	*Lactarius deliciosus*	29.9%	42.1%	0.7%	5.0%
	*Lactarius helvus*	30.8%	11.5%	3.5%	0.0%
Nitrogen heterocycles (NH)	***Cortinarius infractus***	**16.4%**	**56.3%**	**2.6%**	**2.6%**
	*Cortinarius orellanus*	7.3%	9.8%	2.0%	2.5%
	*Lactarius necator*	15.6%	36.0%	4.6%	8.9%
	***Pycnoporus cinnabarinus***	**29.2%**	**106.8%**	**10.9%**	**58.5%**
	*Russula paludosa*	3.5%	18.8%	2.9%	1.8%
	***Russula sardonia***	**35.5%**	**54.2%**	**4.4%**	**6.7%**
Miscellaneous (M)	*Ganoderma adspersum*	16.4%	18.9%	2.3%	4.3%
	***Ganoderma lucidum***	**3.1%**	**75.5%**	**2.5%**	**31.1%**

By comparison of the mean ^1^O_2_-production values of every pathway group, the following ranking could be established: AMP >  > MP > SCP > NH > M for irradiation with blue light (λ = 468 nm), AMP >  > SCP > NH > MP > M for the irradiation with green light (λ = 519 nm) (Fig. 3). Holding true for both irradiation wavelengths, the apolar petroleum ether extracts of the AMP group were the most active ones. In general, the four other pathway groups did not show nearly as high photoactivity as the AMP group. As depicted in Table 1, the PE extracts of *C.* *cinnabarinus* (φ_1O2_|DMA|BL = 296.1%) and *Daldinia concentrica* (φ_1O2_|DMA|BL = 210.6%) were considerably more active than all other extracts when irradiated with blue light. In the green light irradiation group, again the extract prepared from *D. concentrica* fruiting bodies exhibited the most efficient ^1^O_2_-formation (φ_1O2_|DMA|GL = 79.5%), followed by the extracts of *C. cinnamomeoluteus* (φ_1O2_|DMA|GL = 55.2%) and *C. malicorius* (φ_1O2_|DMA|GL = 49.0%).

The polar methanolic extracts of all pathways generally exhibited higher photoactivity compared to the non-polar extracts. Looking at the mean relative ^1^O_2_-production values, the pathway groups could be ranked the same for both irradiation groups with AMP > SCP > NH > MP > M (Fig. 3). Again, fungal extracts containing pigments of the acetate-malonate pathway were the most efficient light-dependent generators of ^1^O_2_. In the blue light irradiation group, the *D. concentrica* extract (φ_1O2_|DMA|BL = 119.9%) as well as extracts prepared from *Cortinarius* species, e.g., *C. olivaceofuscus* (φ_1O2_|DMA|BL = 116.7%) and *C. uliginosus* (φ_1O2_|DMA|BL = 108.2%), were highly active.

When irradiated with blue light, some fungal extracts with pigments from the shikimate-chorismate pathway (SCP) also demonstrated noticeable activity. The PE extracts of *Paxillus atrotomentosus* (φ_1O2_|DMA|BL = 72.4%) and *Gymnopilus penetrans* (φ_1O2_|DMA|BL = 43.6%) marked the outliers with ^1^O_2_-production values close to or above 50% when irradiated with blue light. In the nitrogen heterocycles (NH) group, the methanol extract of *Pycnoporus cinnabarinus* (φ_1O2_|DMA|BL = 106.8%) stood out with a value comparable to that of berberine, besides the extracts of *Cortinarius infractus* (φ_1O2_|DMA|BL = 56.3%) and *Russula sardonia* (φ_1O2_|DMA|BL = 54.2%), which exhibited values of about 50%.

In combination with green light, the methanol extracts of *D. concentrica* (φ_1O2_|DMA|GL = 61.7%), *C. cinnabarinus* (φ_1O2_|DMA|GL = 48.5%), and *C. olivaceofuscus* (φ_1O2_|DMA|GL = 36.4%), all belonging to the AMP group, showed the highest activities. Remarkably, the methanolic extract of *P. cinnabarinus* (φ_1O2_|DMA|GL = 58.5%), identified as an outlier in the NH group, also demonstrated promising green light-dependent ^1^O_2_-production. Moreover, the methanol extract prepared from fruiting bodies of *Ganoderma lucidum* also exhibited photoactivity under both irradiation conditions tested (468 nm: φ_1O2_|DMA|BL = 75.5%, 519 nm: φ_1O2_|DMA|GL = 31.1%).

### Phenolic Content—Folin-Ciocalteu Assay

**Fig. 4:**
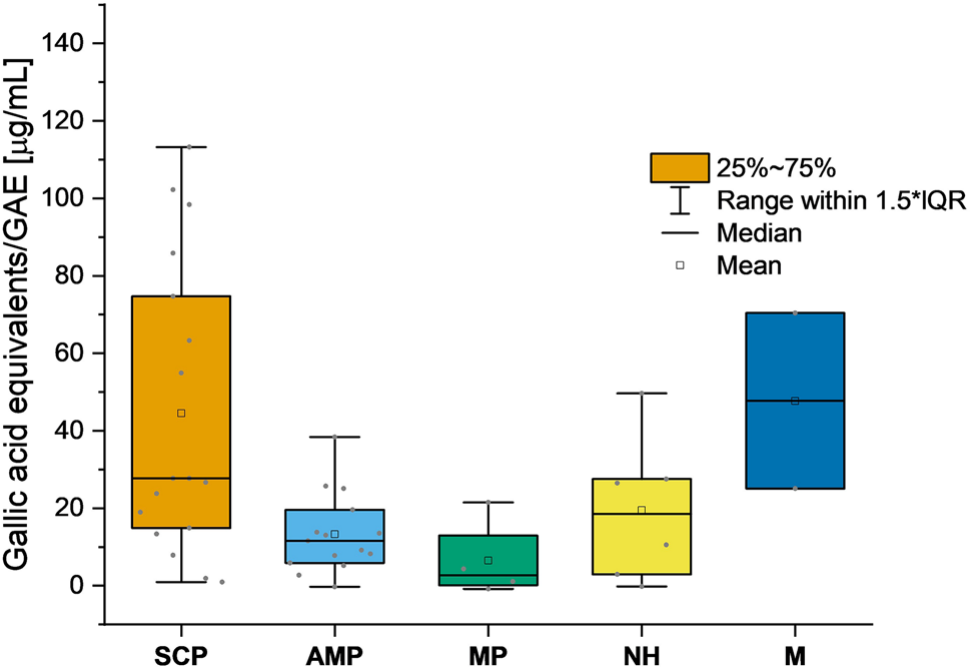
Boxplots representing the phenolic content expressed in gallic acid equivalents/GAE (µg/mL) of the investigated fungal extracts sorted by their affiliation to the major biosynthetic pathways. SCP = shikimate-chorismate pathway; AMP = acetate malonate pathway; MP = mevalonate pathway; NH = nitrogen heterocycles; M = miscellaneous

The total phenolic content of the methanolic fungal extracts was determined using the Folin-Ciocalteu reagent assay [52]. Data analysis was conducted based on descriptive statistics (Fig. 4). Considering the mean phenolic content, expressed in gallic acid equivalents (GAE), the mixed group (i.e., *Ganoderma adspersum* and *G. lucidum*) was found to have the highest mean content (47.7 µg/mL GAE), closely followed by the SCP group (44.5 µg/mL GAE). The mean contents of the AMP and NH groups were comparable with 13.3 and 19.5 µg/mL GAE, respectively. Extracts with pigments derived from the mevalonate pathway (MP) displayed the lowest phenolic contents, resulting in a mean value of 6.5 µg/mL GAE.

**Fig. 5:**
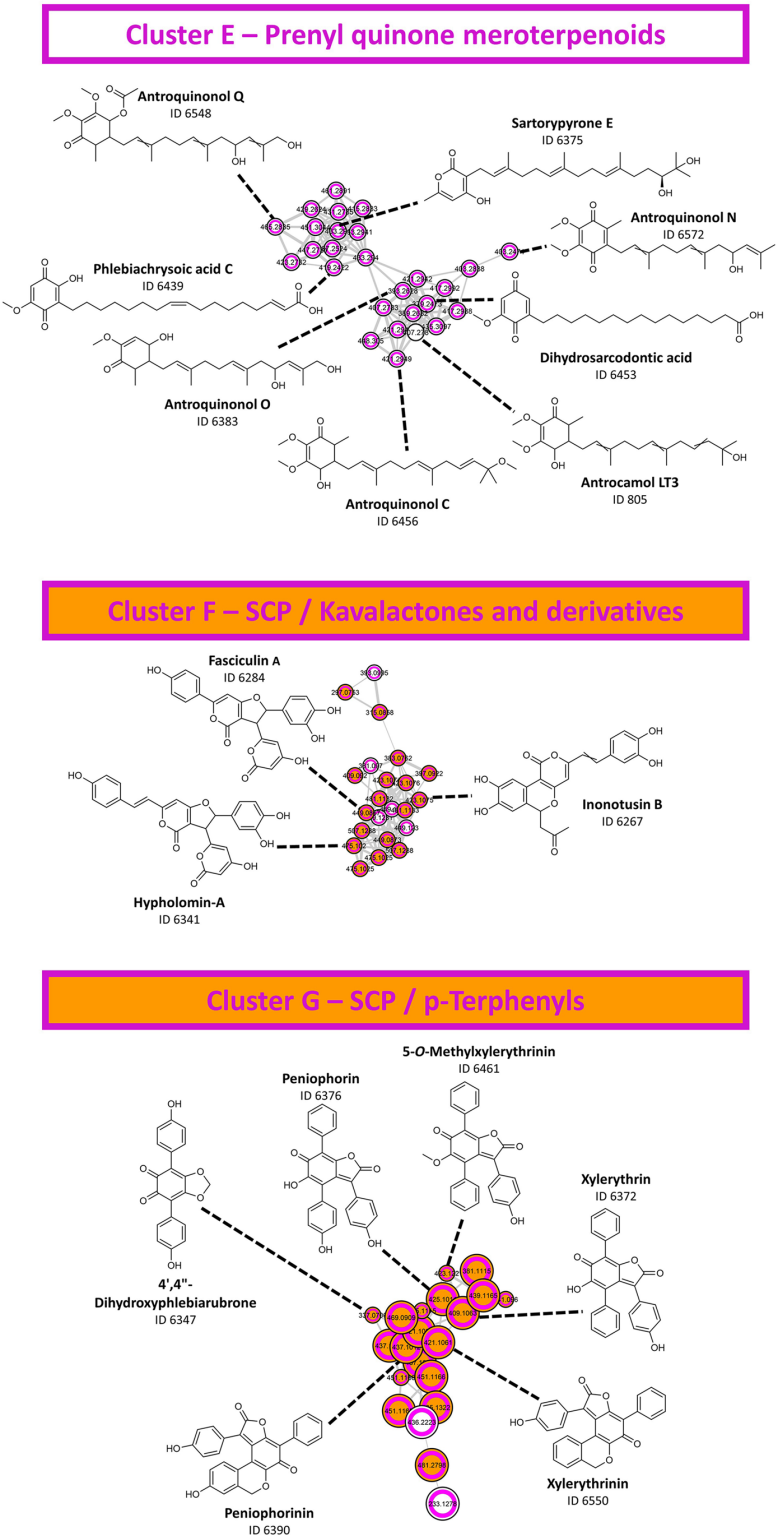
The annotation results for clusters E, F, and G. Features with a high phenolic content were marked with a purple ring. Big nodes represent features with a “VIS-Signal,” whereas small nodes highlight features with an absent "VIS-Signal". The identification level for the given structures is 2. More details of the investigated clusters are provided in the supplementary information

The extracts of *Paxillus atrotomentosus*, *Paxillus involutus*, *Pholiota flammans*, and *Phanerochaete sanguinea* showed the highest phenolic contents with 113.2, 98.4, 102.2, and 74.7 µg/mL GAE, respectively. As these species are known to produce phenolic compounds, such as terphenylquinones, styrylpyrones, and xylerythrins [37], it was interesting to investigate their presence in the tested extracts and the molecular network. Hence, an informational layer based on the Folin-Ciocalteu assay results was added to the FBMN (i.e., extracts with a value of > 50 µg/mL were given a purple ring). Several clusters originating from extracts with a high phenolic content were highlighted and subsequently submitted to further annotation. Cluster E, F, and G (defined in Fig. 2) were putatively identified as clusters containing prenyl quinone meroterpenoids, kavalactones and derivatives, and p-terphenyls, respectively (Fig. 5 and SI Sects. 4.4–4.6).

The features of cluster E, consisting of quinones with extended unbranched/branched side chains, were almost entirely specific to the extract of *Phanerochaete sanguinea*. The presence of these so-called prenyl quinone meroterpenoids has already been described for related *Phanerochaete* species, e.g., for *Phanerochaete chrysosporium* [53].

Cluster F contained features specific to *Pholiota flammans* with dipyrone (i.e., kavalactone) structure. The styrylpyrones hypholomin-A and fasciculin-A are known secondary metabolites of *Pholiota flammans *[54] and were also identified by the utilized annotation workflow. Inonotusin B, a hispidin derivative isolated from *Inonotus hispidus *[55], was likewise putatively annotated for cluster F.

Similar to cluster E, cluster G consisted of features that were primarily found in the methanol extract of *Phanerochaete sanguinea*. Its features were putatively annotated as xylerythrin, 5-O-methylxylerythrin, and peniophorin, the red quinone methide pigments of *Phanerochaete sanguinea* first described by Gripenberg and Martikkala [56, 57].

The comparatively high phenolic contents of extracts prepared from *Ganoderma lucidum* (25.1 µg/mL GAE) and *G. adspersum* (70.4 µg/mL GAE) fruiting bodies are in good agreement with literature data, reporting the presence of, e.g., protocatechuic, p-hydroxybenzoic, and syringic acids in *G. lucidum* [58] and also highlighting their anti-oxidative potential [59, 60].

Interestingly, the extracts with the highest values for ^1^O_2_-production, such as the extracts of *D. concentrica*, *C. olivaceofuscus*, and *C. cinnamomeoluteus*, had only low phenolic contents, with 7.8, 11.6, and 9.2 µg/mL GAE, respectively. In contrast, the highly photoactive methanolic extract of *Pycnoporus cinnabarinus* exhibited a phenolic content slightly higher than *G. lucidum* (27.5 µg/mL GAE). This is most likely due to the presence of the phenolic compound 3-hydroxyanthranilic acid, a potential natural redox mediator of lignin biodegradation, in the ligninolytic polypore [61]. Oxidative coupling of 3-hydroxyanthranilic acid produces cinnabarinic acid, the main pigment of *P. cinnabarinus *[61], exhibiting structural similarity to the known photosensitizer Nile blue [62]. The results, therefore, suggest an interesting interaction between phenolic antioxidants and putative photosensitizing compounds in this polypore. However, further studies are imperative to elucidate the balance between the pro-oxidant effects induced by singlet oxygen and the antioxidant compounds as well as the influence of irradiation on their respective levels present in *P. cinnabarinus* and other fungi.

### (Photo)cytotoxicity Assay

A total of 94 fungal extracts, i.e., 47 petroleum ether and 47 methanol extracts, were tested for their (photo)cytotoxic activity against the three photodynamic therapy (PDT)-relevant cancer cell lines AGS (stomach cancer), T24 (urinary bladder carcinoma), and A549 (non-small cell lung cancer). The in vitro photobiological experiments were conducted using a blue light source (λ = 468 nm ± 27 nm, H = 9.3 J cm^−2^, t_irr_ = 7.5 min) (Table 2). Since all photochemically active extracts could produce ^1^O_2_ under both irradiation conditions (Table 1), the green light source was omitted to achieve high experimental throughput.

**Table 2:**
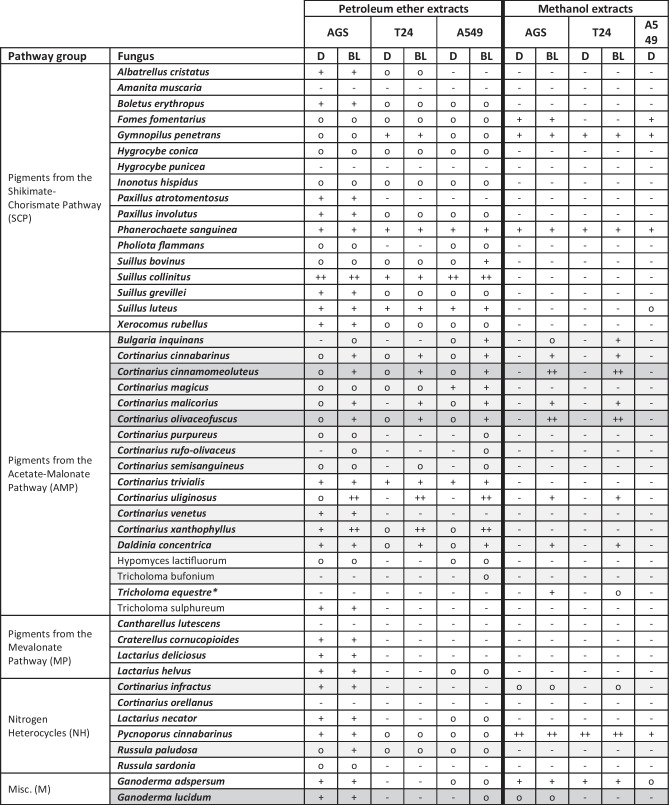
Results of the photobiological evaluation of the fungal extracts based on technical triplicate measurement. (EC_50_ value estimation: ++ … 0.01−5 μg/mL, + … 5−25 μg/mL, o … 25−50 μg/mL, - … >50 μg/mL; Misc. … Miscellaneous, * … dichloromethane instead of methanol extract). Extracts with distinct blue light-dependent toxicity on cancer cells were highlighted in blue, those with weak activity in grey. D … dark, BL … blue light irradiation

The non-polar extracts showed cytotoxicity much more frequently than the polar ones. Furthermore, the extracts could be divided into three groups: (I) extracts without any cytotoxic activity in the tested concentration range, (II) extracts with pronounced dark cytotoxicity (i.e., no light-dependent mode of action), (IIIa) extracts exhibiting weak cytotoxicity after blue-light irradiation, and (IIIb) extracts with pronounced photocytotoxicity.

The group of inactive extracts (I) contained four species, namely *Amanita muscaria*, *Hygrocybe punicea*, *Cantharellus lutescens*, and *Cortinarius orellanus.* The largest group comprised the dark cytotoxic extracts (II), of which many exhibited moderate to high activity (activity level “o” or “ + ,” Table 2). The petroleum ether extracts of *Suillus collinitus* and *S.* *luteus* as well as the two extracts of *Phanerochaete sanguinea* were characterized by strong cytotoxicity against cells of all three cancer cell lines. Unfortunately, the methanolic extract of *Pycnoporus cinnabarinus*, which delivered promising results in the photochemical studies with relative ^1^O_2_-production values of φ_1O2_|DMA|BL = 106.8% and φ_1O2_|DMA|GL = 58.5%, exhibited cytotoxicity independent from light irradiation. Nevertheless, the EC_50_ values for this extract were in the range of 5 µg/mL (A549) or even below (AGS and T24). Recently, Doskocil et al. demonstrated strong cytotoxicity of the *P. cinnabarinus* ethanol extract against Caco-2 (IC_50_ = 81 ± 18 µg/mL) and HT-29 cells (IC_50_ = 31 ± 4 µg/mL) [63]. Therefore, this polypore species is an interesting source for potent cytotoxic agents with potential photosensitizing properties that remain to be thoroughly investigated.

Altogether, fifteen species (31%) were identified as sources of phototoxic extracts. Of these, the extracts of six species exhibited weak photocytotoxicity or indicated possible light-amplified cytotoxicity (IIIa), namely *Cortinarius rufo-olivaceus* (AMP group), *C. semisanguineus* (AMP group), *Russula paludosa* (NH group), *C. infractus* (NH group), *Ganoderma lucidum* (M group), and *Tricholoma bufonium* (AMP group). The weak effect observed could be due to low concentrations of photosensitizing compounds in the respective extracts. To verify the photocytotoxic activity of these fungi, extracts could be prepared from their colored tissues only. For *T. bufonium* the observed weak photocytotoxicity may be due to 7,7’-biphyscion, and thus an artefact, since in fresh fruiting bodies the precursor (i.e., FDM) is mainly present.

The other ten highly photoactive extracts (IIIb) belonged to species producing pigments derived from the AMP. Interestingly, all extracts which proved to be phototoxic in the biological evaluation were also active in the photochemical experiments employing the DMA assay. Since DMA is a probe for ^1^O_2_, this observation excludes the presence of PDT type I reactions (i.e., generation of hydroxyl or superoxide anion radicals). The observed photosensitizing properties of the active extracts are, therefore, most likely based on type II reactions (i.e., generation of ^1^O_2_) [64]. Overall, *Cortinarius* was the predominant genus with seven out of ten highly active species whose extracts were able to kill cancer cells after irradiation in the low µg/mL-range (e.g., *C. olivaceofuscus* and *C. cinnamomeoluteus*).

Several *Cortinarius* species have already been studied by us in terms of their phototoxic properties, which even led to the isolation and structure elucidation of novel photosensitizers (e.g., 7,7′-biphyscion, dermolutein, and emodin-1-O-β-D-glucopyranoside) [9, 10, 12].

**Fig. 6:**
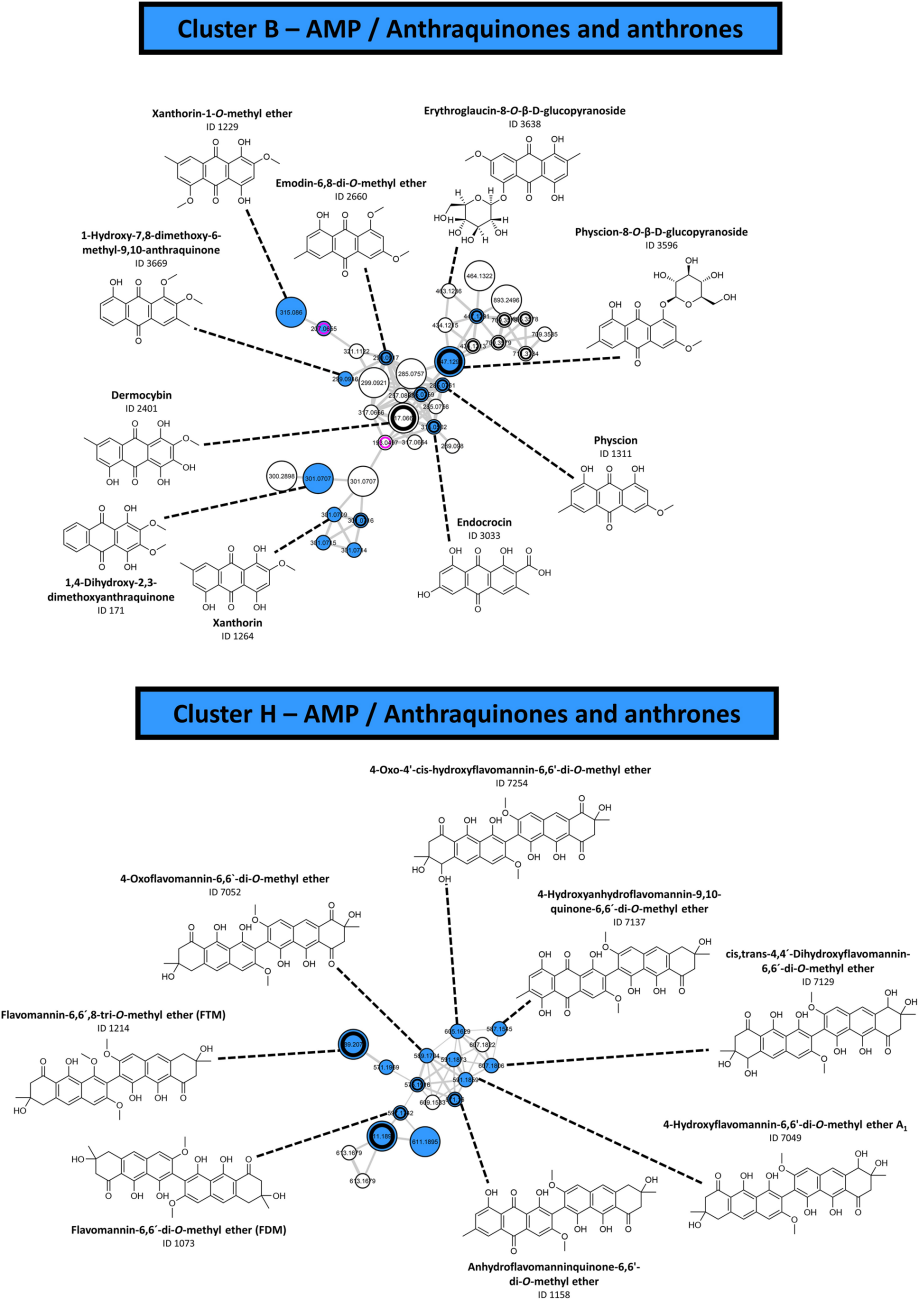
The annotation results for clusters B and H. Features with a high photocytotoxicity against cancer cells were highlighted with a black ring. Big nodes represent features with a “VIS-Signal,” whereas small nodes highlight features with an absent “VIS-Signal.” The identification levels for the given structures is 2. The supplementary information provides more details on both clusters

Exploration of the underlying photocytotoxic principle was done by combining a FBMN-layer highlighting features from photocytotoxic extracts (i.e., active features were given with a black ring) with the layer of the “VIS-Signal” variable. The filter variable “VIS-Signal” is imperative for the detection of photosensitizing compounds, as it visualizes the main requirement of a photosensitizer according to the first law of photochemistry (i.e., the Grotthus-Draper law), namely the absorption of light [30]. Several clusters with features of the AMP with a detectable absorption in the visible spectral range were identified. Interestingly, all these clusters with photoactive features (i.e., clusters A, B, H, and I as defined in Fig. 2) were putatively identified as clusters with anthraquinones and related structures. The features of both clusters, cluster A (SI Sect. 4.2) and cluster I (SI Sect. 4.8), were mainly annotated as anthraquinone carboxylic acids (e.g., cluster A: dermolutein (ID 1047), cinnalutein (ID 2369), and nordermorubin (ID 1121); cluster I: cinnarubin (ID 2363), dermorubin (ID 1246), and cardinalic acid (ID 1036)). Cluster B was found to contain various monomeric anthraquinones (Fig. 6) such as dermocybin (ID 2401), xanthorin (ID 1264), and physcion (ID 1311). Furthermore, cluster H (Fig. 6), containing features from the *Cortinarius* sp. and *Tricholoma* sp. extracts, exhibited various pre-anthraquinones (i.e., the biosynthetic precursors of dimeric anthraquinones). The annotation results for Cluster H present a possible explanation for the photocytotoxic activity of the *Tricholoma equestre* dichloromethane extract. This species is described as a source of dimeric (pre)anthraquinones, such as the photocytotoxic 7,7′-biphyscion [37].

Overall, these findings are in line with our previous reports on photoactive/cytotoxic extracts of fruiting bodies of *Cortinarius* species. Hence, the identification of fungal anthraquinones as markers of photoactivity, done in the framework of an in-depth FBMN-study of the genus *Cortinarius*, could be further validated in this study [30]. As the classical *Cortinarius* subgenus *Dermocybe* has repeatedly been shown to be rich in photosensitizing compounds, further metabolic and mycochemical studies of the largely unexplored South American and Australian *Dermocybe* species could potentially provide an even more comprehensive picture. The combination of metabolomics and phylogenetics could also offer insights into the evolution of secondary metabolites in the subgenus *Dermocybe*.

In addition, the extracts of *D. concentrica* (AMP) and *Bulgaria inquinans* (AMP) were also able to induce cell death when combined with blue light irradiation. In contrast to most ascomycetes, these two species produce comparatively large sporulating structures: *B. inquinans* apothecia reach a diameter of 0.5–4 cm, the ball-shaped stromata with perithecia of *D. concentrica* reach a diameter of 2–7 cm. The methanol extracts of these two ascomycetes, which form characteristic black fruiting bodies, showed promising photocytotoxicity in the preliminary study in a concentration range of only 5–25 µg/mL, without exhibiting pronounced dark cytotoxicity. This is the first report of photocytotoxicity for these two species.

**Fig. 7:**
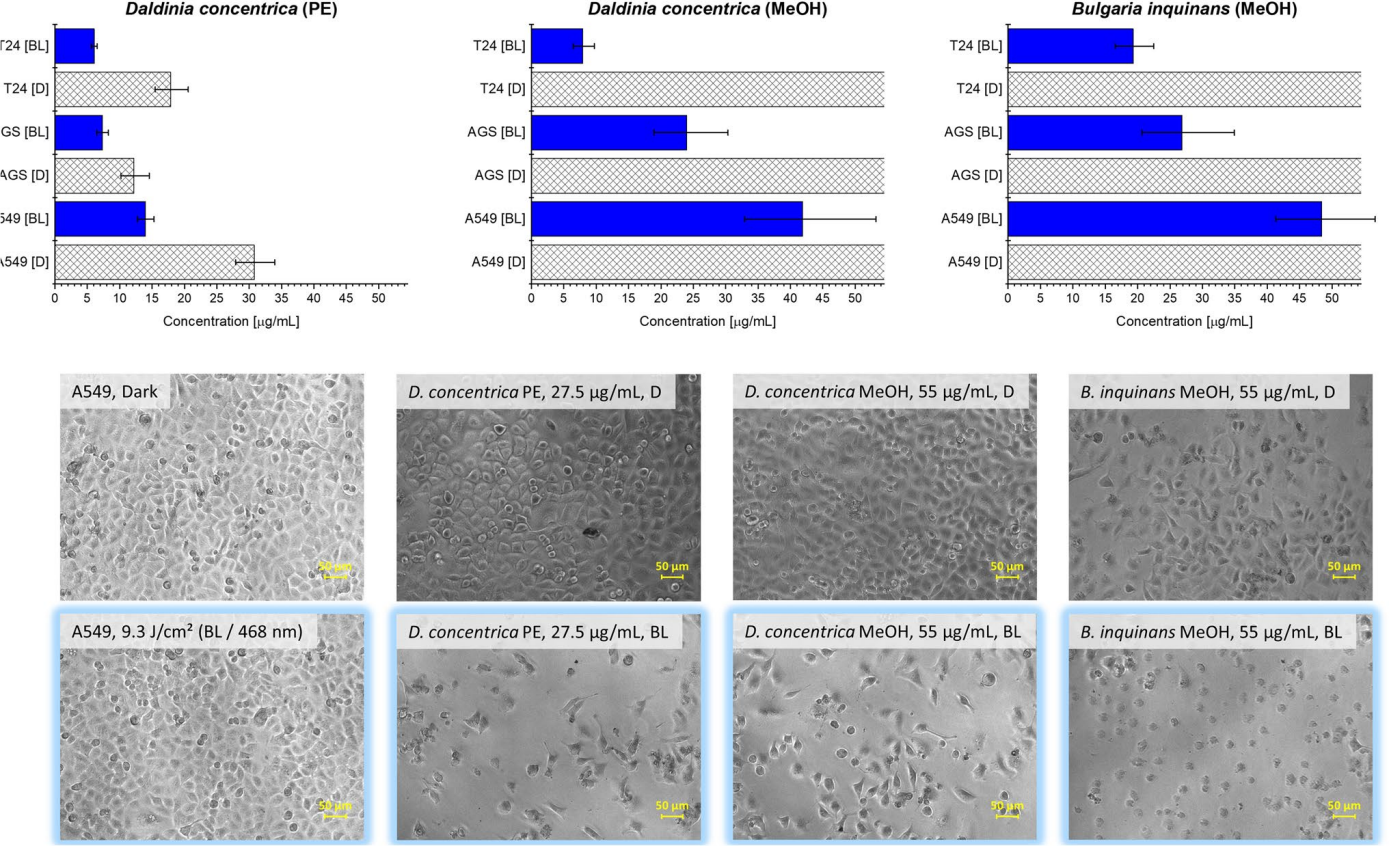
**A** Results of the photobiological evaluation of the extracts of *D. concentrica* (PE and MeOH) and *B. inquinans* (MeOH) depicted as bar plots. EC_50_ values were calculated on the basis of triplicate measurement (i.e., 3 × 3 technical replicates) using six testing concentrations. Irradiation parameters were chosen as follows: BL/blue light, λ = 468 ± 27 nm, 9.3 J/cm.^2^, blue bars. Dark cytotoxicity (D) was determined with the same testing concentrations in the absence of any light source. **b** Micrographs (200 × magnification) of the A549 non-small cell lung cancer cell line in the presence of the tested extracts (top line: dark control group; bottom line: irradiated group, blue colored border)

Since the extracts of *D. concentrica* (PE and MeOH) and *B. inquinans* (MeOH) afforded interesting and novel insights, they were subjected to further biological experiments. Once again, they were examined for their blue light-dependent cytotoxic activity on the three cancer cell lines A549, AGS, and T24 (Fig. 7a, b). This time, however, the analysis was performed in biological triplicate with additional testing concentrations (i.e., 0.55, 2.75, 5.50, 11.00, 27.50, and 55.00 µg/mL) for the calculation of EC_50_ values and photoindices. Blue light activation led to a drastic increase in photocytotoxicity for all three extracts, as indicated by their photoindices (P.I. > 1).

The *D. concentrica* PE extract demonstrated strong dark toxicity, resulting in photoindices ranging from 1.7 to 3.0. Several studies of the phytochemical profile of *D. concentrica* resulted in the isolation of moderately cytotoxic compounds such as daldinin (IC_50_|MCF7 = 13.5 ± 1.2 µM) or 6-hydroxymellein (IC_50_|MCF7 = 494.2 µM), [65, 66], which could explain the observed cytotoxicity in the dark. The *D. concentrica* and *B. inquinans* methanol extracts did not show any cytotoxicity in the absence of light in the tested concentration range. Blue light irradiation, however, led to moderate cytotoxicity for the *B. inquinans* extract with EC_50_ values of 48.4, 26.8, and 19.3 µg/mL against A549, AGS, and T24 cells, respectively. The most promising results gave the *D. concentrica* methanol extract with low EC_50_ values (i.e., EC_50_|A549|BL = 41.9 µg/mL, EC_50_|AGS|BL = 24.0 µg/mL, and EC_50_|T24|BL = 7.9 µg/mL) as well as good photoindices. A comprehensive list of EC_50_ values and photoindices is given in the supplementary information (SI Sect. 5). Structural identification of molecular clusters in the FBMN associated with the photocytotoxicity of *B. inquinans* and *D. concentrica*, however, was not possible with the obtained data. Even though several clusters, e.g., cluster C and cluster D, could be clearly linked to photocytotoxic extracts and exhibited a positive “VIS-Signal” variable, the annotation workflow failed to yield meaningful structural suggestions. As stated before, this is probably due to the lack of experimental MS^2^ data of fungal pigments. *B. inquinans* and *D. concentrica* are both described to produce pigments of the acetate-malonate pathway, i.e., polyketides, which are potentially responsible for the observed photocytotoxicity. Exhibiting structural similarities with known photosensitizers, e.g., with the perylenequinone cercosporin from *Cercospora kikuchii* [67, 68] and with the napththodianthrone hypericin from *Hypericum perforatum* [69], the quinones bulgarein and bulgarhodin represent potentially interesting polyketides occurring in *B. inquinans.*

The related compound 4,9-dihydroxyperylene-3,10-quinone is described for both species [70]. In 2013, Li and colleagues were able to isolate two new anthraquinone dimers from the fruiting bodies of *B. inquinans*, namely bulgareone A and bulgareone B [71]. With the discovery of several photosensitizing dimeric anthraquinones with promising anticancer, antiparasitic, or even antiviral activity, such as 7,7′-biphyscion from *C. uliginosus* [10] and 5,5′-bisoranjidiol from *Heterophyllaeae lycioides* [72, 73], the investigation of the bulgareones with respect to a photodynamic effect could be of great interest. Thus, taking together the high levels of singlet oxygen production and the considerable light-dependent photocytotoxicity of the extracts of *B. inquinans* and *D. concentrica*, their thorough myco- and photochemical analysis could yield highly active compounds with extraordinary value for the field of photodynamic therapy.

## Conclusions

By investigating a set of 48 fruiting body-forming fungal species, we were able to show on a metalevel that photoactivity is common in fungi. FBMN analysis with additional bioactivity layers and the VIS signal proved to be a convenient tool to visualize the complex conglomerate of fungi’s secondary metabolites and phototoxicity. Nevertheless, the experimental logic is not limited to fungal extracts; other neglected sources, like bacteria or marine sponges, can be systematically explored.

Our results showed that photochemical activity occurs in many different fungi from various genera. Extracts containing pigments from all major metabolic pathways, i.e., shikimate-chorismate, acetate-malonate, mevalonate pathway, and nitrogen heterocycles, were able to produce singlet oxygen. Furthermore, most of these extracts triggered—in a blue light-dependent fashion—cell death. Nevertheless, photoactivity is concentrated in fungal species producing pigments of the acetate-malonate pathway. Besides species of the genus *Cortinarius*, the fruit bodies of the two ascomycetes *Bulgaria inquinans* and *Daldinia concentrica* were identified as promising sources for novel natural photosensitizers. Next to these, the results of the fruiting bodies of *Russula paludosa* and *Ganoderma lucidum* indicated photobiological activity. In sum, we could show that photoactivity is a trait discoverable in each major metabolic pathway yielding pigments and thus these results hint further towards a hidden photochemical defense mechanism in the kingdom Fungi.

## Supplementary Information

Below is the link to the electronic supplementary material.

Supplementary file1 (DOCX 5544 KB)

## Data Availability

The datasets generated during and/or analyzed during the current study are available in the Massive repository. Link: https://massive.ucsd.edu/ProteoSAFe/static/massive.jsp): MSV000088756 [10.25345/C5DC69]. All other datasets generated and analyzed during the current study are available from the corresponding author on reasonable request.
