# Supplementary material for: Highlighting the Phototherapeutical Potential of Fungal Pigments in Various Fruiting Body Extracts with Informed Feature-Based Molecular Networking
Source: Microb Ecol. 2023 Mar 22;86(3):1972–92. doi: 10.1007/s00248-023-02200-2 (PMC10497435; doi:10.1007/s00248-023-02200-2)
Supplement: Supplementary file 1 — Supplementary file1 (DOCX 5544 KB) [file 248_2023_2200_MOESM1_ESM.docx]

Electronic supplementary information (SI)

Highlighting the photopharmacological potential of fungal pigments in various fruiting body extracts with informed feature-based molecular networking^†^

Fabian Hammerle^1^, Luis Quirós-Guerrero^2,3^, Jean-Luc Wolfender^2,3^, Ursula Peintner^4^, Bianka Siewert^1*^

^1^ Mag.pharm. F. J. Hammerle, Dr. B. Siewert, Institute of Pharmacy/Pharmacognosy, Center for Molecular Biosciences Innsbruck (CMBI), University of Innsbruck, Innrain 80/82, 6020 Innsbruck, Austria.

^2^ M.Sc. L. Quirós-Guerrero, Prof. Dr. J.-L. Wolfender, Phytochemistry and Bioactive Natural Products, School of Pharmaceutical Sciences, University of Geneva, CMU - Rue Michel-Servet 1, 1211 Geneva, Switzerland.

^3^ M.Sc. L. Quirós-Guerrero, Prof. Dr. J.-L. Wolfender, Institute of Pharmaceutical Sciences of Western Switzerland, University of Geneva, CMU, 1211 Geneva, Switzerland.

^4^ Prof. Dr. U. Peintner, Institute of Microbiology, University of Innsbruck, Technikerstrasse 25d, 6020 Innsbruck, Austria.

*Corresponding author, Email: [bianka.siewert@uibk.ac.at](mailto:bianka.siewert@uibk.ac.at)

†Electronic Supplementary Information (SI) available: Origin of fungal biomaterial, photochemical data (DMA-assay), Folin-Ciocalteu assay data, (photo)cytotoxicity evaluation, and feature-based molecular networking.

Table of Contents

[2 Fungal material 2](#_Toc126511682)

[3 Extract yields 4](#_Toc126511683)

[4 Feature-based molecular networking (FBMN) – Cluster annotation 5](#_Toc126511684)

[4.1 Overview of selected clusters 5](#_Toc126511685)

[4.2 Cluster A 6](#_Toc126511686)

[4.3 Cluster B 8](#_Toc126511687)

[4.4 Cluster E 10](#_Toc126511688)

[4.5 Cluster F 12](#_Toc126511689)

[4.6 Cluster G 14](#_Toc126511690)

[4.7 Cluster H 16](#_Toc126511691)

[4.8 Cluster I 18](#_Toc126511692)

[5 (Photo)biological evaluation 20](#_Toc126511693)

[6 Micrographs (48 hours after irradiation) – *B. inquinans*, *D. concentrica* 21](#_Toc126511694)

# Fungal material

Voucher material of the investigated fungal species is either deposited in the mycological collection of the Tiroler Landesmuseen (IBF) or in the Verein für Pilzkunde München e.V. (VPM) (<http://www.pilze-muenchen.de/index.html>).

**Table S1.** Fungal species (in alphabetical order) used in this study with their respective voucher numbers and collection data.

| **Species** | **Voucher** | **leg. et det.** | **Origin** |
| --- | --- | --- | --- |
| *Albatrellus cristatus* (Schaeff.) Kotl. & Pouzar | IBF20170592 | B. Siewert | Italy, Emilia-Romagna, Monte Penna |
| *Amanita muscaria* (L.) Lam. | IBF20170587 | B. Siewert | Italy, Emilia-Romagna, Bedonia, Prato Ballerino |
| *Boletus erythropus* Pers. | IBF20190127 | F. Bellu´ | Italy, Emilia-Romagna, Bedonia |
| *Bulgaria inquinans* (Pers.) Fr. | VPM1802 | H. Grünert | Germany, Bavaria, Eichsee |
| *Cantharellus lutescens* Fr. | IBF20180156 | R. Zollitsch | China, Market in Dali |
| *Cortinarius cinnabarinus* Fr. | IBF19801005 | R. Pöder | Sweden, Småland, Femsjö |
| *Cortinarius cinnamomeoluteus* P.D. Orton | IBF19710420 | M. Moser | Swiss, Luzern |
| *Cortinarius infractus* (Pers.) Fr. | IBF20190146 | U. Peintner | Italy, Bedonia |
| *Cortinarius magicus* Eichhorn | IBF20170491 | F. Bellu´ | Italy, Emilia-Romagna, Bedonia, Albareto |
| *Cortinarius malicorius* Fr. | IBF2018009 | D. Borghi | Italy, South Tyrol |
| *Cortinarius olivaceofuscus* Kühner  (*D. carpineti* nom. inval.) | IBF19750023 | M. Moser | Switzerland, Basel |
| *Cortinarius orellanus* Fr. | IBF20190149 | U. Peintner | Italy, Bedonia |
| *Cortinarius phoeniceus* (Bull.) R. Maire (C. purpureus (Bull. ex Pers.) Bidaud, Moënne-Locc. & Reumaux) | IBF19750022 | M. Moser | Germany, Baden-Württemberg |
| *Cortinarius rufo-olivaceus* (Pers.) Fr. | IBF20190113 | F. Bellu´ | Italy, Emilia-Romagna, Parma, Borgotaro |
| *Cortinarius semisanguineus (Fr.) Gillet* | IBF19740665 | M. Moser | Sweden, Småland, Femsjö |
| *Cortinarius trivialis* J.E. Lange | IBF20170586 | U. Peintner | Italy, Massa Carrara Passo del Bratello |
| *Cortinarius uliginosus* Berk. | IBF19951120 | M. Moser | Austria, Tyrol |
| *Cortinarius venetus* (Fr.) Fr. | IBF20180233 | U. Peintner | Italy, Orto Botanico Forestale dell'Abetone |
| *Cortinarius xanthophyllus* (Cooke) Rob. Henry | IBF20190109 | F. Bellu´ | Italy, Emilia-Romagna, Parma, Borgotaro |
| *Craterellus cornucopioides* (L.) Pers. | IBF20170588 | B. Siewert | Italy, Emilia-Romagna, Bedonia, Prato Ballerino |
| *Cytidia salicina* (Fr.) Burt | VPM2011 | B. Fellmann | Germany, Bavaria, Spitzingsee |
| *Daldinia concentrica* (Bolton) Ces. & De Not. | VPM741 | P. Karasch | Germany, Bavaria, Weilheim |
| *Fomes fomentarius* (L.) Fr. | IBF20130019 | U. Peintner | Austria, Tyrol, Innsbruck |
| *Ganoderma adspersum* (Schulzer) Donk | IBF20190129 | U. Peintner | Italy, Bedonia |
| *Ganoderma lucidum* (Curtis) P. Karst. | IBF20180157 | B. Siewert | PLANTASIA GmbH (Nr. 680898) |
| *Gymnopilus penetrans* (Fr.) Murrill | IBF20170587 | B. Siewert | Italy, Emilia-Romagna, Bedonia, Prato Ballerino |
| *Hygrocybe conica* (Schaeff.) P. Kumm. | IBF20050017 | U. Peintner | Austria, Tyrol |
| *Hygrocybe punicea* (Fr.) P. Kumm. | IBF20170078 | U. Peintner | Austria, Tyrol |
| *Hypomyces lactifluorum* (Schwein.) Tul. | IBF20200079 | P. L’Archeveque | Canada |
| *Inonotus hispidus* (Bull.) P. Karst. | IBF20170590 | B. Siewert | Italy, Emilia-Romagna, Bedonia, Prato Ballerino |
| *Lactarius deliciosus* (L.) Gray | IBF19740555 | B. Cetto | Italy, Trento |
| *Lactarius helvus* (Fr.) Fr. | IBF19800473 | M. Moser | Austria, Tyrol |
| *Lactarius necator* (Bull.) Pers. | IBF19780218 | M. Moser | Sweden, Femsjo |
| *Paxillus atrotomentosus* (Batsch) Fr. | IBF19720105 | M. Moser | Sweden, Femsjo |
| *Paxillus involutus* (Batsch) Fr. | IBF20100097 | U. Peintner | Italy, Southtyrol, Kurzras |
| *Phanerochaete sanguinea* (Fr.) Pouzar | IBF19970716 | U. Peintner | Austria, Styria, Grebenzen |
| *Pholiota flammans* (Batsch) P. Kumm. | IBF20170591 | M.E. Salgado Salomon | Italy, Emilia-Romagna, Monte Penna |
| *Pycnoporus cinnabarinus* (Jacq.) P. Karst. | IBF20180010 | B. Siewert | Austria, Innsbruck, Hungerburg |
| *Russula paludosa* Britzelm. | IBF20100032 | U. Peintner | Austria, Tyrol |
| *Russula sardonia* Fr. | IBF19980133 | M. Moser | Sweden, Femsjo |
| *Suillus bovinus* (L.) Roussel | IBF20060143 | U. Peintner | Austria, Tyrol |
| *Suillus collinitus* (Fr.) Kuntze | IBF20010719 | U. Peintner | Italy, Abbruzzo, Opi |
| *Suillus grevillei (Klotzsch) Singer* | IBF20070074 | U. Peintner | Italy, Southtyrol, Prettau |
| *Suillus luteus* (L.) Roussel | IBF20170053 | U. Peintner | Austria, Tyrol |
| *Tricholoma bufonium* (Pers.) Gillet | IBF20190139 | U. Peintner | Italy, Emilia-Romagna, Monte Penna |
| *Tricholoma equestre* (L.) P. Kumm. | IBF20190154 | H. Grünert | Italy, Emilia-Romagna, Monte Penna |
| *Tricholoma sulphureum* (Bull.) P. Kumm. | IBF20190155 | U. Peintner | Italy, Emilia-Romagna, Monte Penna |
| *Xerocomus rubellus* Quél. | IBF20030037 | U. Peintner | Italy, Emilia-Romagna, Monte Penna |

# Extract yields

**Table S2.** Overview of the extract yields (PE … petroleum ether, MeOH … methanol) obtained with different extraction methods (i.e., Soxhlet extraction and ultra-sonication).

| **Soxhlet extraction** | **Mass biomaterial [g]** | **Yield [mg (%dw)]** | |
| --- | --- | --- | --- |
|  |  | **PE extract** | **MeOH extract** |
| *Albatrellus cristatus* | 2.00 | 25.0 (1.3%) | 340.1 (17.0%) |
| *Amanita muscaria* | 2.07 | 103.3 (5.0%) | 770.0 (37.2%) |
| *Boletus erythropus* | 2.46 | 53.0 (2.2%) | 890.3 (36.2%) |
| *Bulgaria inquinans* | 2.26 | 34.3 (1.5%) | 450.2 (19.9%) |
| *Cantharellus lutescens* | 2.08 | 130.0 (6.3%) | 589.7 (28.4%) |
| *Cortinarius cinnabarinus* | 2.08 | 37.0 (1.8%) | 775.0 (37.2%) |
| *Cortinarius cinnamomeoluteus* | 2.11 | 30.4 (1.4%) | 643.6 (30.5%) |
| *Cortinarius infractus* | 0.89 | 15.4 (1.7%) | 292.3 (32.8%) |
| *Cortinarius magicus* | 1.97 | 16.0 (0.8%) | 499.0 (25.3%) |
| *Cortinarius malicorius* | 2.08 | 22.0 (1.1%) | 666.0 (31.9%) |
| *Cortinarius olivaceofuscus* | 2.06 | 15.0 (0.7%) | 687.0 (33.3%) |
| *Cortinarius orellanus* | 0.25 | 5.9 (2.4%) | 86.0 (34.4%) |
| *Cortinarius phoeniceus* | 1.98 | 50.0 (2.5%) | 884.0 (44.8%) |
| *Cortinarius rufo-olivaceus* | 2.34 | 86.2 (3.7%) | 789.5 (33.7%) |
| *Cortinarius semisanguineus* | 2.08 | 33.0 (1.6%) | 750.0 (36.0%) |
| *Cortinarius trivialis* | 2.01 | 34.7 (1.7%) | 499.6 (24.9%) |
| *Cortinarius uliginosus* | 2.13 | 51.0 (2.4%) | 797.0 (37.4%) |
| *Cortinarius venetus* | 2.02 | 38.6 (1.9%) | 726.5 (36.0%) |
| *Cortinarius xanthophyllus* | 2.04 | 27.6 (1.4%) | 599.4 (29.4%) |
| *Craterellus cornucopioides* | 0.90 | 17.2 (1.9%) | 275.1 (30.6%) |
| *Daldinia concentrica* | 0.72 | 12.3 (1.7%) | 197.9 (27.5%) |
| *Fomes fomentarius* | 2.47 | 16.9 (0.7%) | 63.6 (2.6%) |
| *Ganoderma adspersum* | 2.30 | 69.9 (3.0%) | 234.1 (10.2%) |
| *Gymnopilus penetrans* | 2.01 | 20.5 (1.0%) | 669.6 (33.3%) |
| *Hygrocybe conica* | 1.10 | 109.4 (9.9%) | 365.0 (33.2%) |
| *Hygrocybe punicea* | 2.50 | 98.9 (4.0%) | 448.4 (17.9%) |
| *Inonotus hispidus* | 2.03 | 15.4 (0.8%) | 402.0 (19.8%) |
| *Lactarius deliciosus* | 2.83 | 51.2 (1.8%) | 760.7 (26.9%) |
| *Lactarius helvus* | 2.84 | 35.4 (1.2%) | 684.1 (24.1%) |
| *Lactarius necator* | 2.08 | 84.4 (4.1%) | 679.0 (32.6%) |
| *Paxillus atrotomentosus* | 2.31 | 48.1 (2.1%) | 679.4 (29.4%) |
| *Paxillus involutus* | 2.54 | spilled | 830.5 (32.7%) |
| *Paxillus involutus* | 0.70 | 23.4 (3.3%) | 223.7 (32.0%) |
| *Phanerochaete sanguinea* | 0.40 (large wood content) | 6.1 (1.5%) | 57.9 (14.5%) |
| *Pholiota flammans* | 2.04 | 21.3 (1.0%) | 672.4 (33.0%) |
| *Pycnoporus cinnabarinus* | 2.02 | 18.6 (0.9%) | 127.1 (6.3%) |
| *Russula paludosa* | 2.55 | 83.9 (3.3%) | 1060.4 (41.6%) |
| *Russula sardonia* | 2.59 | 131.6 (5.1%) | 844.4 (32.6%) |
| *Suillus bovinus* | 1.45 | 24.5 (1.7%) | 488.5 (33.7%) |
| *Suillus collinitus* | 2.02 | 59.5 (2.9%) | 713.0 (35.3%) |
| *Suillus grevillei* | 2.50 | 59.6 (2.4%) | 890.2 (35.6%) |
| *Suillus luteus* | 2.20 | 66.1 (3.0%) | 812.7 (36.9%) |
| *Xerocomus rubellus* | 2.04 | 42.7 (2.1%) | 560.3 (27.5%) |
| **Ultra-sonication** |  |  |  |
| *Cytidia salicina* | 7.50E-3 | / | 1.2 (16.0%) |
| *Ganoderma lucidum* | 7.16 | 23.7 (0.3%) | 228.4 (3.2%) |
| *Hypomyces lactifluorum* | 7.71 | 58.4 (0.8%) | 1260.8 (16.4%) |
| *Tricholoma bufonium* | 1.04 | 41.9 (4.0%) | 211.4 (20.3%) |
| *Tricholoma equestre* | 59.0 | 1824.8 (3.1%) | 1148.2 (1.9%) |
| *Tricholoma sulphureum* | 0.83 | 29.5 (3.6%) | 103.5 (12.5%) |

# Feature-based molecular networking (FBMN) – Cluster annotation

## Overview of selected clusters


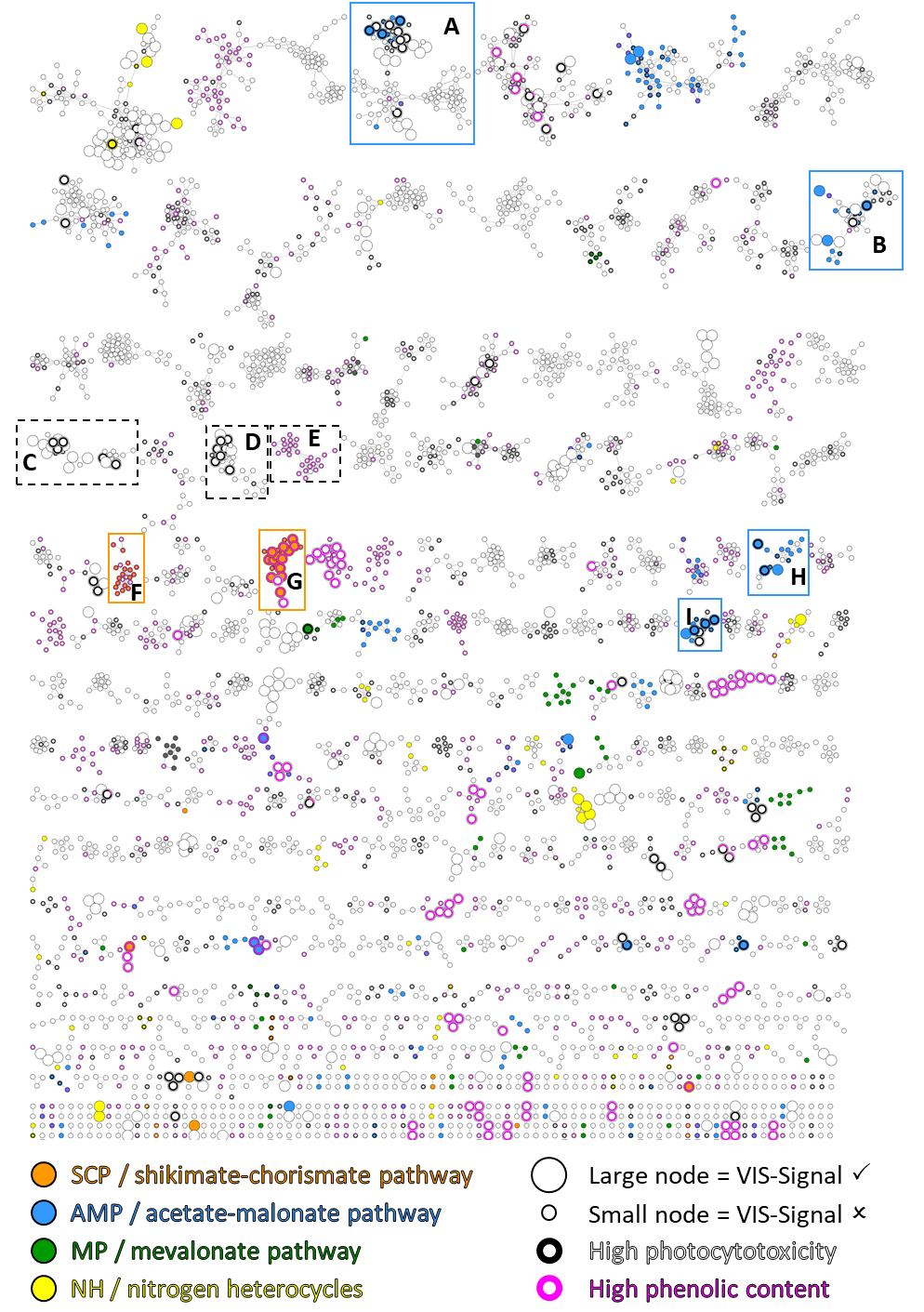


**Figure S1.** Feature-based molecular network visualized with Cytoscape. The illustration code is given in the lower part of the figure. Clusters subjected to annotation beyond the “class” level or discussed in the manuscript are lettered A-I.

## Cluster A


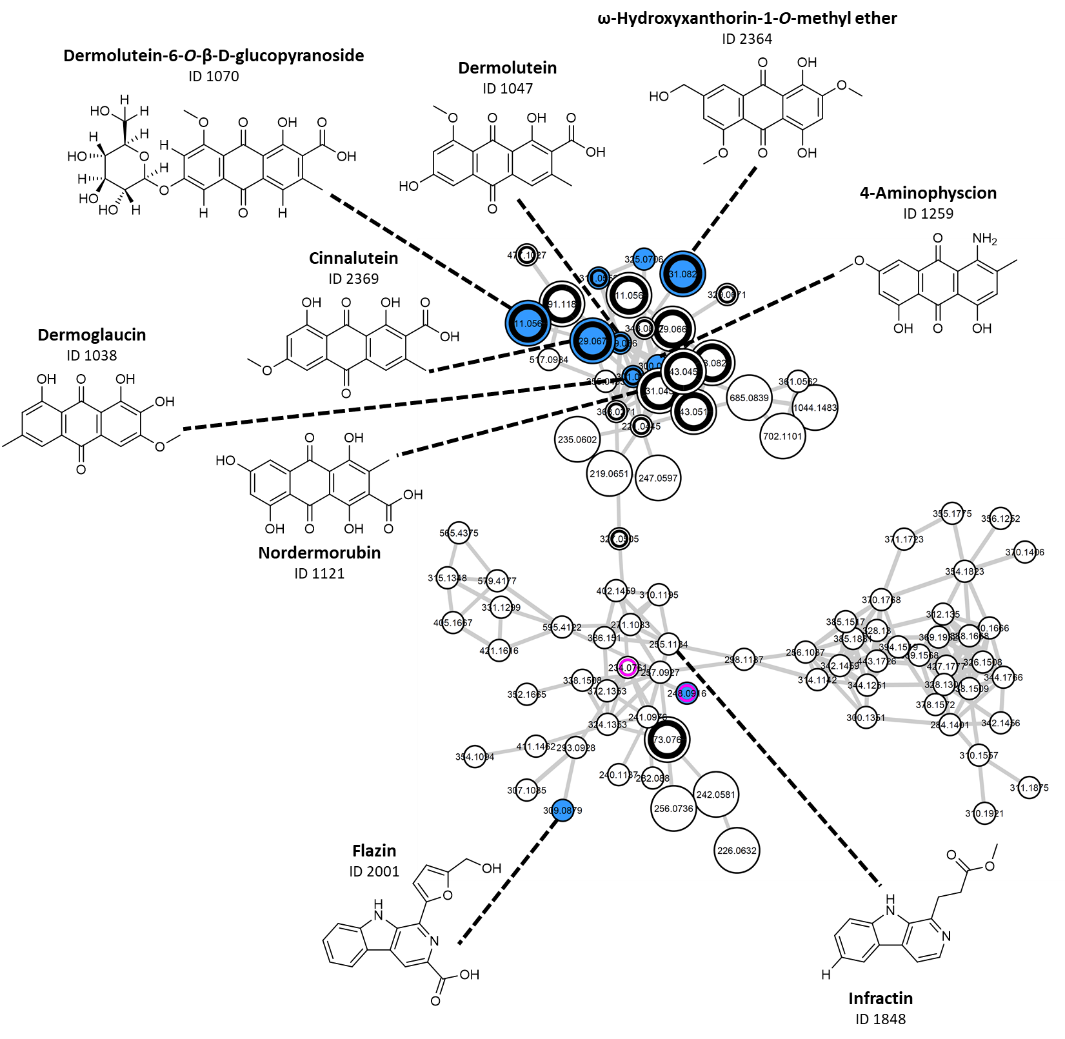


**Figure S2.** Annotated Cluster A. Each node displays the precursor mass (please zoom in). Out of the different molecule annotations listed in the table below (Table S3), the chemical structure was depicted, which was deemed most probable. Illustration code: blue node filling … acetate-malonate pathway, large node … “VIS-Signal” present, small node … “VIS-Signal” absent, black ring … high photocytotoxicity, pink ring … high phenolic content.

**Table S3.** The annotation results for Cluster A presented as the respective compound ID (shared name), the “VIS-Signal”, m/z, the molecular formula, the SMILES (top 🡪 bottom: GNPS, In-house library, ISDB-DNP; Black color … SMILES belonging to the chemical structure drawn in the figure, Grey color … additional hits, Green color … identical output, / … no hit at all), the name, and the NPClassifier “class”.

| **Compound ID (shared name)** | **"VIS-Signal" (yes = 1/no = 0)** | **m/z** | **Molecular formula** | **Annotation (SMILES): GNPS \| In-house library \| ISDB-DNP** | **Name [Identification level]** | **NPClassifier "class"** |
| --- | --- | --- | --- | --- | --- | --- |
| 2369 | 1 | 329.0678 | C17H12O7 | / | Cinnalutein [2] | Flavones |
|  |  |  |  | O=C1C2=C(C=C(C)C(C(O)=O)=C2O)C(C3=CC(OC)=CC(O)=C31)=O |  |  |
|  |  |  |  | O=C(O)Cc1cc(O)cc2oc(-c3ccc(O)c(O)c3)cc(=O)c12 |  |  |
| 1259 | 0 | 300.0866 | C16H13NO5 | / | 4-Aminophyscion [2] | Anthraquinones and anthrones |
|  |  |  |  | O=C1C2=C(O)C=C(OC)C=C2C(C3=C1C(O)=CC(C)=C3N)=O |  |  |
|  |  |  |  | COc1cc(O)c2c(c1)C(=O)c1c(N)c(C)cc(O)c1C2=O |  |  |
| 2001 | 0 | 309.0879 | C17H12N2O4 | / | Flazin [2] | Aporphine alkaloids |
|  |  |  |  | OCC1=CC=C(O1)C2=NC(C(O)=O)=CC3=C2NC4=CC=CC=C43 |  |  |
|  |  |  |  | COc1c2c3c(ccnc3c3c(O)cccc13)C(=O)C(=O)N2C |  |  |
| 1848 | 0 | 255.1134 | C15H14N2O2 | / | Infractin [2] | / |
|  |  |  |  | [H]C1=CC=C(NC2=C3C=CN=C2CCC(OC)=O)C3=C1 |  |  |
|  |  |  |  | / |  |  |
| 1047 | 0 | 329.066 | C17H12O7 | / | Dermolutein [1, isolated compound] | Anthraquinones and anthrones |
|  |  |  |  | O=C1C2=C(C=C(C)C(C(O)=O)=C2O)C(C3=CC(O)=CC(OC)=C31)=O |  |  |
|  |  |  |  | COc1cc(O)cc2c1C(=O)c1c(cc(C)c(C(=O)O)c1O)C2=O |  |  |
| 1121 | 1 | 331.0453 | C16H10O8 | / | Nordermorubin [2] | / |
|  |  |  |  | O=C1C2=C(O)C=C(O)C=C2C(C3=C1C(O)=C(C(O)=O)C(C)=C3O)=O |  |  |
|  |  |  |  | / |  |  |
| 1038 | 0 | 301.071 | C16H12O6 | / | Dermoglaucin [2] | Anthraquinones and anthrones |
|  |  |  |  | O=C1C2=C(C=C(C)C=C2O)C(C3=CC(OC)=C(O)C(O)=C31)=O |  |  |
|  |  |  |  | COc1cc2c(c(O)c1O)C(=O)c1c(O)cc(C)cc1C2=O |  |  |
| 2364 | 1 | 331.0826 | C17H14O7 | / | ω-Hydroxyxanthorin-1-*O*-methyl ether [2] | Aflatoxins |
|  |  |  |  | O=C1C2=C(O)C=C(OC)C(O)=C2C(C3=C1C(OC)=CC(CO)=C3)=O |  |  |
|  |  |  |  | COc1cc2c(c3oc(=O)c4c(c13)CCC4=O)C1CC(O)OC1O2 |  |  |
| 1070 | 1 | 491.1188 | C23H22O12 | / | Dermolutein-6-*O*-β-D-glucopyranosid [2] | / |
|  |  |  |  | OC(C(C(O)=O)=C(C([H])=C1C(C2=C3C(OC)=C([H])C(O[C@@]4([H])[C@] ([C@@]([C@]([C@](C([H])([H])O)([H])O4)([H])O)([H])O)([H])O)=C2[H])=O)C)=C1C3=O |  |  |
|  |  |  |  | / |  |  |

## Cluster B


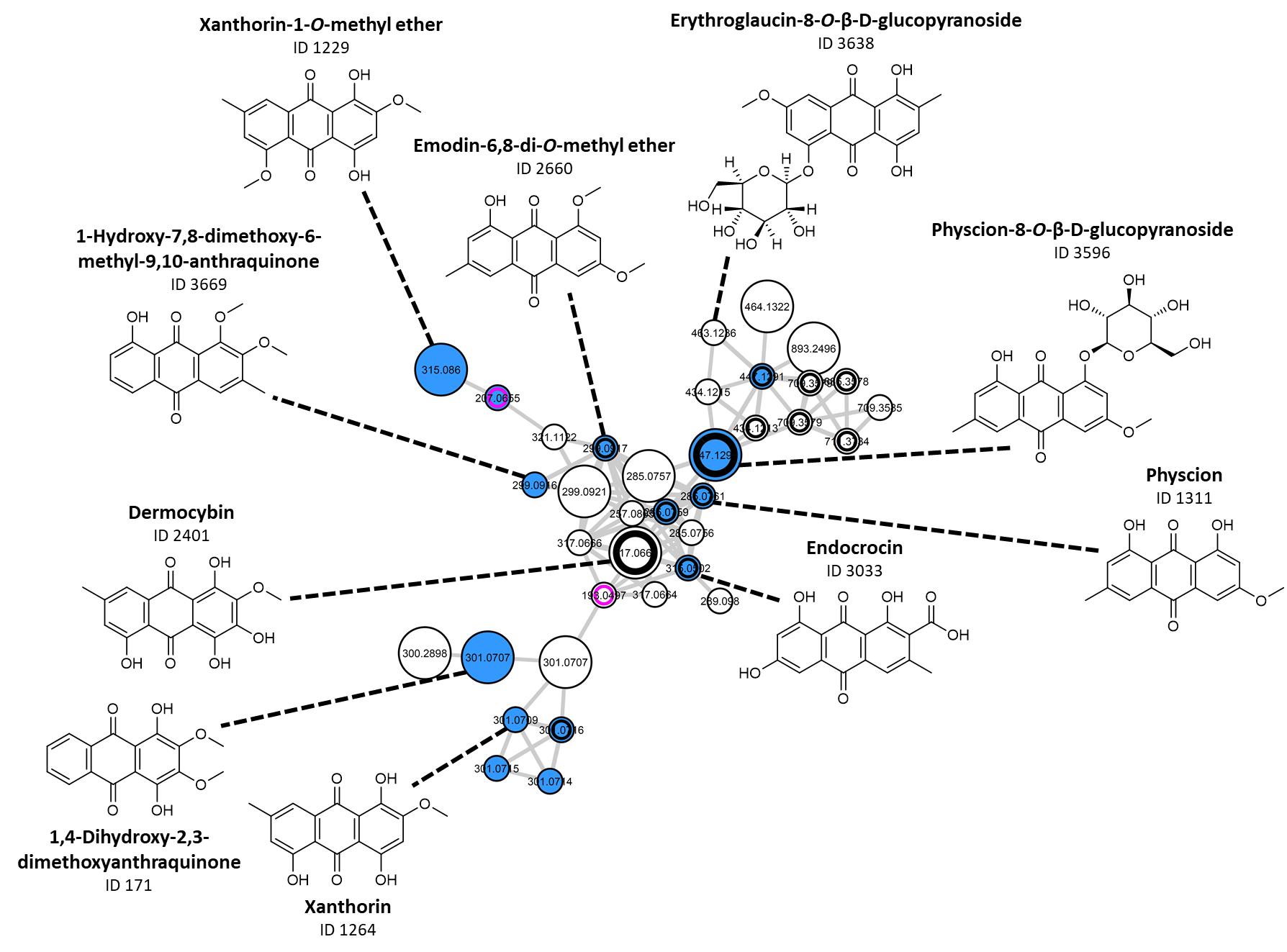


**Figure S3.** Annotated Cluster B. Each node displays the precursor mass (please zoom in). Out of the different molecule annotations listed in the table below (Table S4), the chemical structure was depicted, which was deemed most probable. Illustration code: blue node filling … acetate-malonate pathway, large node … “VIS-Signal” present, small node … “VIS-Signal” absent, black ring … high photocytotoxicity, pink ring … high phenolic content.

**Table S4.** The annotation results for Cluster B presented as the respective compound ID (shared name), the “VIS-Signal”, m/z, the molecular formula, the SMILES (top 🡪 bottom: GNPS, In-house library, ISDB-DNP; Black color … SMILES belonging to the chemical structure drawn in the figure, Grey color … additional hits, Green color … identical output, / … no hit at all), the name, and the NPClassifier “class”.

| **Compound ID (shared name)** | **"VIS-Signal" (yes = 1/no = 0)** | **m/z** | **Molecular formula** | **Annotation: GNPS (Name) \| In-house library (SMILES) \| ISDB-DNP (SMILES)** | **Name [Identification level]** | **NPClassifier "class"** |
| --- | --- | --- | --- | --- | --- | --- |
| 2401 | 1 | 317.0665 | C16H12O7 | Spectral Match to Tamarixetin from NIST14 | Dermocybin [2] | / |
|  |  |  |  | O=C1C2=C(C=C(C)C=C2O)C(C3=C(O)C(OC)=C(O)C(O)=C31)=O |  |  |
|  |  |  |  | / |  |  |
| 3033 | 0 | 315.0502 | C16H10O7 | 3-Methylquercetin | Endocrocin [2] | Anthraquinones and anthrones |
|  |  |  |  | O=C1C2=C(C=C(C)C(C(O)=O)=C2O)C(C3=CC(O)=CC(O)=C31)=O |  |  |
|  |  |  |  | Cc1cc2c(c(O)c1C(=O)O)C(=O)c1c(O)cc(O)cc1C2=O |  |  |
| 171 | 1 | 301.0707 | C16H12O6 | Geraldol | 1,4-Dihydroxy-2,3-dimethoxyanthraquinone [2] | Anthraquinones and anthrones |
|  |  |  |  | O=C1C2=C(O)C=C(OC)C=C2C(C3=C1C(O)=CC(CO)=C3)=O |  |  |
|  |  |  |  | COc1c(O)c2c(c(O)c1OC)C(=O)c1ccccc1C2=O |  |  |
| 1311 | 0 | 285.0761 | C16H12O5 | 5,7-Dihydroxy-6-methoxy-2-phenylchromen-4-one | Physcion [2] | Anthraquinones and anthrones |
|  |  |  |  | O=C1C2=C(O)C=C(OC)C=C2C(C3=C1C(O)=CC(C)=C3)=O |  |  |
|  |  |  |  | COc1cc(O)c2c(c1)C(=O)c1cc(C)cc(O)c1C2=O |  |  |
| 1264 | 0 | 301.0709 | C16H12O6 | Geraldol | Xanthorin [2] | Anthraquinones and anthrones |
|  |  |  |  | O=C1C2=C(O)C=C(OC)C(O)=C2C(C3=C1C(O)=CC(C)=C3)=O |  |  |
|  |  |  |  | COc1cc(O)c2c(c1O)C(=O)c1cc(C)cc(O)c1C2=O |  |  |
| 3669 | 0 | 299.0916 | C17H14O5 | 2-[(2,5-Dimethoxyphenyl)methylene]benzo[b]thiophen-3-one | 1-Hydroxy-7,8-dimethoxy-6-methyl-9,10-anthraquinone [2] | Anthraquinones and anthrones |
|  |  |  |  | OC1=C2C(C(C(C=C(C)C=C3OC)=C3C2=O)=O)=CC(OC)=C1 |  |  |
|  |  |  |  | COc1c(C)cc2c(c1OC)C(=O)c1c(O)cccc1C2=O |  |  |
| 2660 | 0 | 299.0917 | C17H14O5 | 2-[(2,5-Dimethoxyphenyl)methylene]benzo[b]thiophen-3-one | Emodin-6,8-di-*O*-methylether [2] | Anthraquinones and anthrones |
|  |  |  |  | O=C1C2=C(OC)C=C(OC)C=C2C(C3=C1C(O)=CC(C)=C3)=O |  |  |
|  |  |  |  | COc1cc(OC)c2c(c1)C(=O)c1cc(C)cc(O)c1C2=O |  |  |
| 3596 | 1 | 447.1291 | C22H22O10 | 3-(4-hydroxyphenyl)-7-methoxy-5-[(3R,4S,5S,6R)-3,4,5-trihydroxy-6-(hydroxymethyl)oxan-2-yl]oxychromen-4-one | Physcion-8-*O*-β-D-glucopyranoside [2] | Anthraquinones and anthrones |
|  |  |  |  | O=C1C2=C(O[C@@]([H])([C@](O)3[H])O[C@]([H])(CO)[C@@] ([H])(O)[C@@]3(O)[H])C=C(OC)C=C2C(C4=C1C(O)=CC(C)=C4)=O |  |  |
|  |  |  |  | COc1cc(O[C@@H]2O[C@H](CO)[C@@H](O)[C@H](O)[C@H]2O)c2c(c1)C(=O)c1cc(C)cc(O)c1C2=O |  |  |
| 3638 | 0 | 463.1236 | C22H22O11 | Spectral Match to Peonidin 3-O-glucoside cation from NIST14 | Erythroglaucin-8-*O*-β-D-glucopyranoside [2] | / |
|  |  |  |  | O=C1C2=C(O[C@@]([H])([C@](O)3[H])O[C@]([H])(CO)[C@@] ([H])(O)[C@@]3(O)[H])C=C(OC)C=C2C(C4=C1C(O)=CC(C)=C4O)=O |  |  |
|  |  |  |  | / |  |  |
| 1229 | 1 | 315.086 | C17H14O6 | Cirsimaritin | Xanthorin-1-*O*-methylether [2] | Anthraquinones and anthrones |
|  |  |  |  | O=C1C2=C(O)C=C(OC)C(O)=C2C(C3=C1C(OC)=CC(C)=C3)=O |  |  |
|  |  |  |  | COc1cc(O)c2c(c1O)C(=O)c1cc(C)cc(OC)c1C2=O |  |  |

## Cluster E


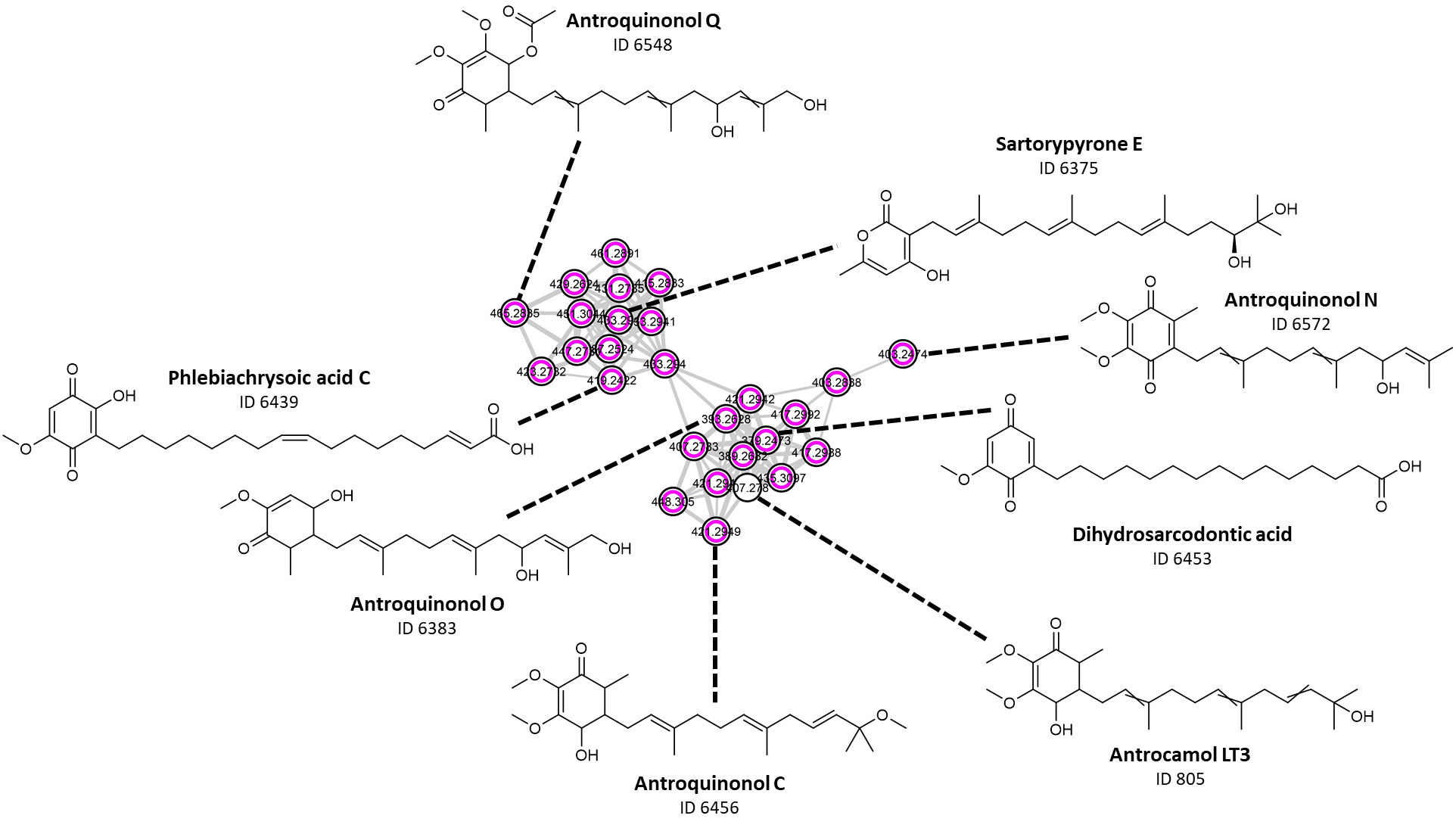


**Figure S4.** Annotated Cluster E. Each node displays the precursor mass (please zoom in). Out of the different molecule annotations listed in the table below (Table S5), the chemical structure was depicted, which was deemed most probable. Illustration code: large node … “VIS-Signal” present, small node … “VIS-Signal” absent, pink ring … high phenolic content.

**Table S5.** The annotation results for Cluster E presented as the respective compound ID (shared name), the “VIS-Signal”, m/z, the molecular formula, the SMILES (top 🡪 bottom: GNPS, In-house library, ISDB-DNP; Black color … SMILES belonging to the chemical structure drawn in the figure, Grey color … additional hits, Green color … identical output, / … no hit at all), the name, and the NPClassifier “class”.

| **Compound ID (shared name)** | **"VIS-Signal" (yes = 1/no = 0)** | **m/z** | **Molecular formula** | **Annotation (SMILES): GNPS \| In-house library \| ISDB-DNP** | **Name [Identification level]** | **NPClassifier "class"** |
| --- | --- | --- | --- | --- | --- | --- |
| 6375 | 0 | 433.294 | C26H40O5 | / | Sartorypyrone E [2] | Prenyl quinone meroterpenoids |
|  |  |  |  | / |  |  |
|  |  |  |  | C/C(=C\CC/C(C)=C/Cc1c(O)cc(C)oc1=O)CC/C=C(\C)CC[C@H](O)C(C)(C)O |  |  |
| 805 | 0 | 407.278 | C24H38O5 | / | Antrocamol LT3 [2] | Prenyl quinone meroterpenoids |
|  |  |  |  | / |  |  |
|  |  |  |  | COC1=C(OC)C(O)C(CC=C(C)CCC=C(C)CC=CC(C)(C)O)C(C)C1=O |  |  |
| 6456 | 0 | 421.2949 | C25H40O5 | / | Antroquinonol C [2] | Prenyl quinone meroterpenoids |
|  |  |  |  | / |  |  |
|  |  |  |  | COC1=C(OC)C(O)C(C/C=C(\C)CC/C=C(\C)C/C=C/C(C)(C)OC)C(C)C1=O |  |  |
| 6453 | 0 | 379.2473 | C22H34O5 | / | Dihydrosarcodontic acid [2] | Ergostane steroids |
|  |  |  |  | COC1=CC(C=C(CCCCCCCCCCCCCCC(O)=O)C1=O)=O |  |  |
|  |  |  |  | C/C(=C/C1OC(=O)C(C)C1C)C1CCC2C3CC(=O)OC3CCC12C |  |  |
| 6383 | 0 | 393.2628 | C23H36O5 | / | Antroquinonol O [2] | Prenyl quinone meroterpenoids |
|  |  |  |  | / |  |  |
|  |  |  |  | COC1=CC(O)C(C/C=C(\C)CC/C=C(\C)CC(O)/C=C(\C)CO)C(C)C1=O |  |  |
| 6548 | 0 | 465.2835 | C26H40O7 | / | Antroquinonol Q [2] | Prenyl quinone meroterpenoids |
|  |  |  |  | / |  |  |
|  |  |  |  | COC1=C(OC)C(OC(C)=O)C(CC=C(C)CCC=C(C)CC(O)C=C(C)CO)C(C)C1=O |  |  |
| 6572 | 0 | 403.2474 | C24H34O5 | / | Antroquinonol N [2] | Prenyl quinone meroterpenoids |
|  |  |  |  | / |  |  |
|  |  |  |  | COC1=C(OC)C(=O)C(CC=C(C)CCC=C(C)CC(O)C=C(C)C)=C(C)C1=O |  |  |
| 6439 | 0 | 419.2422 | C24H34O6 | / | Phlebiachrysoic acid C [2] | Phthalide derivatives |
|  |  |  |  | O=C(C(OC)=C1)C(CCCCCCC/C=C\CCCCC/C=C/C(O)=O)=C(O)C1=O |  |  |
|  |  |  |  | COc1cc(OC)c2c(c1)C(CCCCCCCCCC(=O)CCC(C)=O)OC2=O |  |  |

## Cluster F


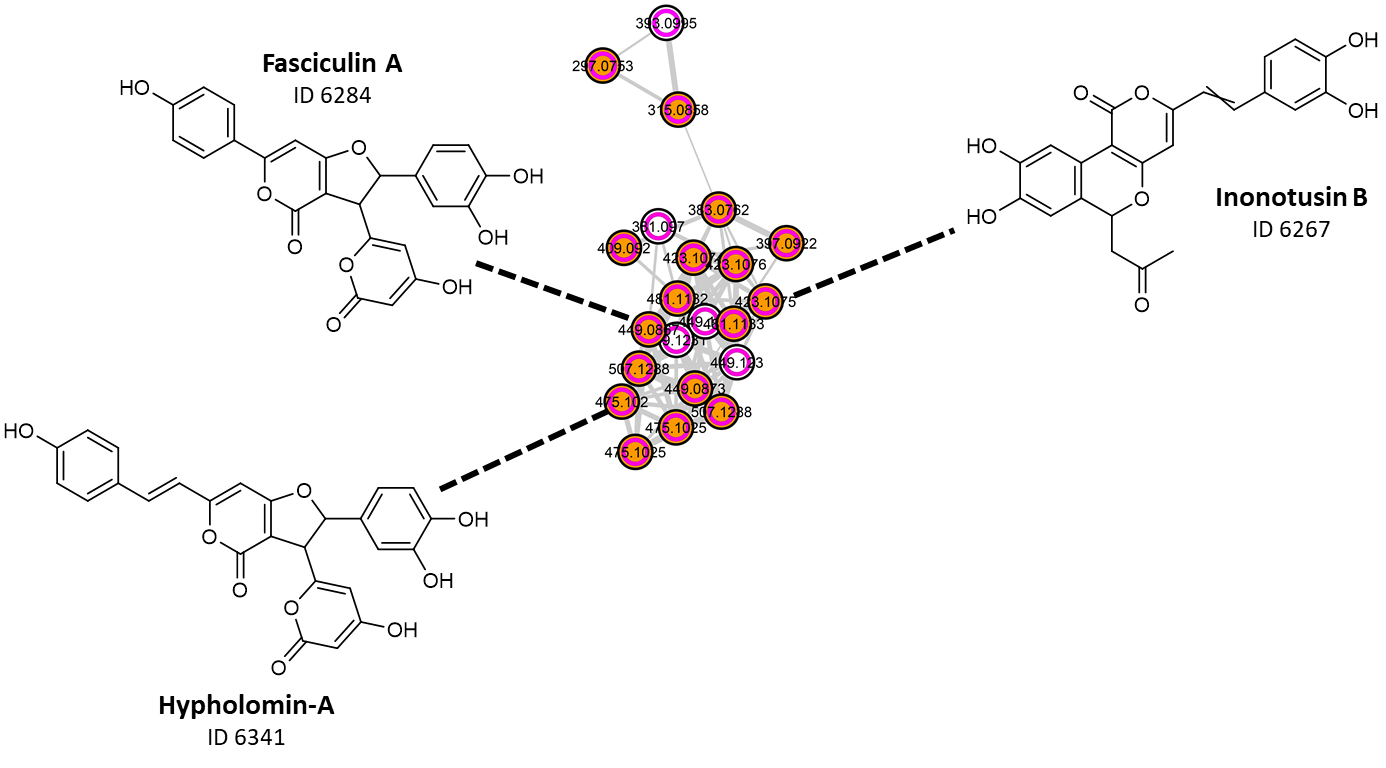


**Figure S5.** Annotated Cluster F. Each node displays the precursor mass (please zoom in). Out of the different molecule annotations listed in the table below (Table S6), the chemical structure was depicted, which was deemed most probable. Illustration code: orange node filling … shikimate-chorismate pathway, large node … “VIS-Signal” present, small node … “VIS-Signal” absent, pink ring … high phenolic content.

**Table S6.** The annotation results for Cluster F presented as the respective compound ID (shared name), the “VIS-Signal”, m/z, the molecular formula, the SMILES (top 🡪 bottom: GNPS, In-house library, ISDB-DNP; Black color … SMILES belonging to the chemical structure drawn in the figure, Grey color … additional hits, Green color … identical output, / … no hit at all), the name, and the NPClassifier “class”.

| **Compound ID (shared name)** | **"VIS-Signal" (yes = 1/no = 0)** | **m/z** | **Molecular formula** | **Annotation (SMILES): GNPS \| In-house library \| ISDB-DNP** | **Name [Identification level]** | **NPClassifier "class"** |
| --- | --- | --- | --- | --- | --- | --- |
| 6267 | 0 | 423.1075 | C23H18O8 | / | Inonotusin B [2] | Kavalactones and derivatives |
|  |  |  |  | OC1=CC=C(/C=C(C2=C(O)C=C(/C=C/C3=CC=C(O)C(O)=C3)OC2=O)\C(C)=O)C=C1O |  |  |
|  |  |  |  | CC(=O)CC1Oc2cc(C=Cc3ccc(O)c(O)c3)oc(=O)c2-c2cc(O)c(O)cc21 |  |  |
| 6284 | 0 | 481.1133 | C24H16O9 | / | Fasciculin-A [2] | Kavalactones and derivatives |
|  |  |  |  | O=C1OC(C2=CC=C(O)C=C2)=CC3=C1C(C(O4)=CC(O)=CC4=O)C(C5=CC=C(O)C(O)=C5)O3 |  |  |
|  |  |  |  | O=C1OC(C2=CC=C(O)C=C2)=CC3=C1C(C(O4)=CC(O)=CC4=O)C(C5=CC=C(O)C(O)=C5)O3 |  |  |
| 6341 | 0 | 475.102 | C26H18O9 | / | Hypholomin-A [2] | Kavalactones and derivatives |
|  |  |  |  | OC(C=C1)=CC=C1/C=C/C(O2)=CC3=C(C(C(O4)=CC(O)=CC4=O)C(C5=CC=C(O)C(O)=C5)O3)C2=O |  |  |
|  |  |  |  | OC(C=C1)=CC=C1/C=C/C(O2)=CC3=C(C(C(O4)=CC(O)=CC4=O)C(C5=CC=C(O)C(O)=C5)O3)C2=O |  |  |

## Cluster G


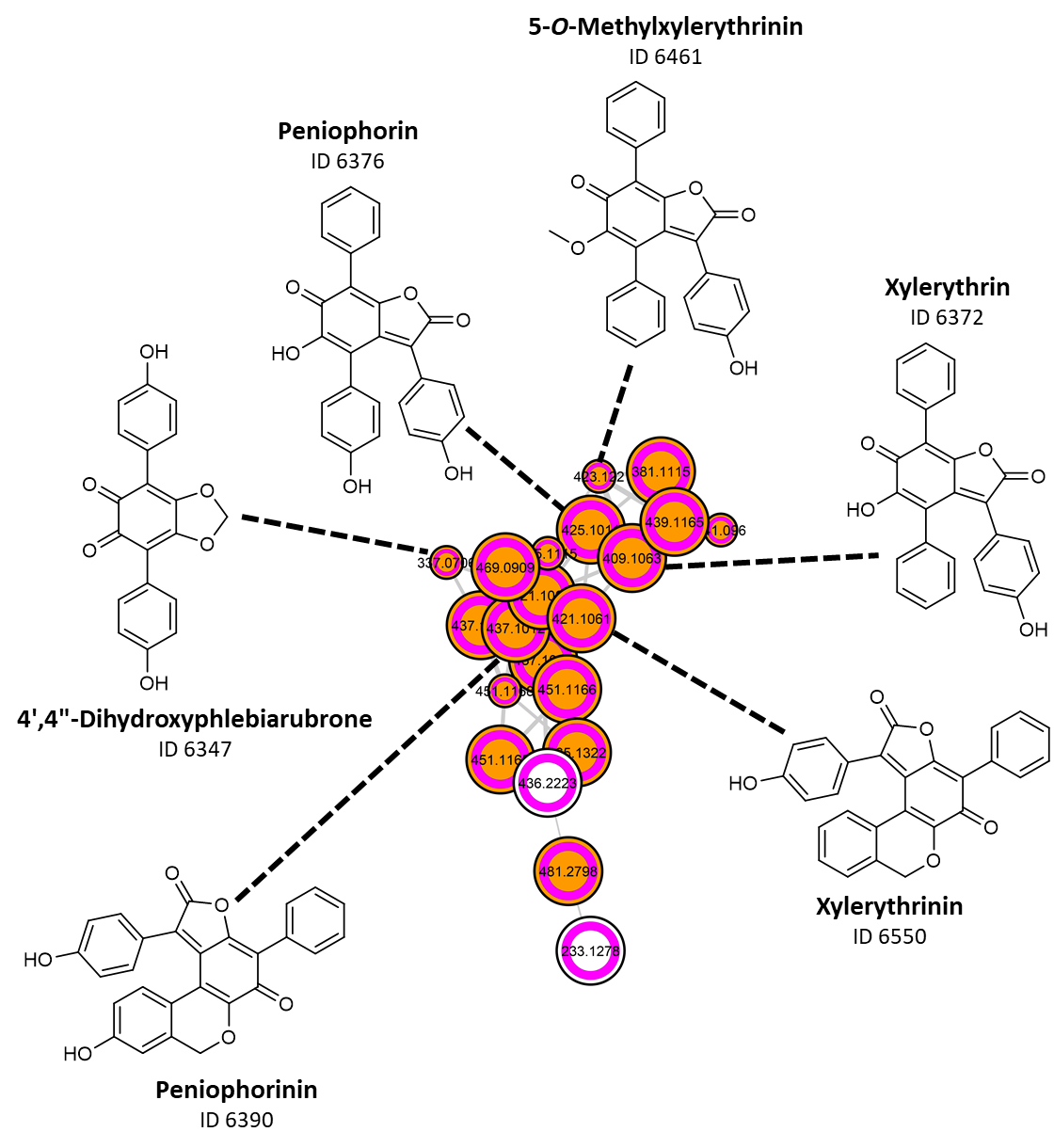


**Figure S6.** Annotated Cluster G. Each node displays the precursor mass (please zoom in). Out of the different molecule annotations listed in the table below (Table S7), the chemical structure was depicted, which was deemed most probable. Illustration code: orange node filling … shikimate-chorismate pathway, large node … “VIS-Signal” present, small node … “VIS-Signal” absent, pink ring … high phenolic content.

**Table S7.** The annotation results for Cluster G presented as the respective compound ID (shared name), the “VIS-Signal”, m/z, the molecular formula, the SMILES (top 🡪 bottom: GNPS, In-house library, ISDB-DNP; Black color … SMILES belonging to the chemical structure drawn in the figure, Grey color … additional hits, Green color … identical output, / … no hit at all), the name, and the NPClassifier “class”.

| **Compound ID (shared name)** | **"VIS-Signal" (yes = 1/no = 0)** | **m/z** | **Molecular formula** | **Annotation (SMILES): GNPS \| In-house library \| ISDB-DNP** | **Name [Identification level]** | **NPClassifier "class"** |
| --- | --- | --- | --- | --- | --- | --- |
| 6550 | 1 | 421.1061 | C27H16O5 | / | Xylerythrinin [2] | p-Terphenyls |
|  |  |  |  | O=C1C(C2=CC=C(O)C=C2)=C(C(O1)=C3C4=CC=CC=C4)C(C5=C6C=CC=C5)=C(OC6)C3=O |  |  |
|  |  |  |  | O=C1C(C2=CC=C(O)C=C2)=C(C(O1)=C3C4=CC=CC=C4)C(C5=C6C=CC=C5)=C(OC6)C3=O |  |  |
| 6347 | 0 | 337.0706 | C19H12O6 | / | 4',4"-Dihydroxyphlebiarubrone [2] | p-Terphenyls |
|  |  |  |  | O=C1C(C2=CC=C(O)C=C2)=C(OCO3)C3=C(C4=CC=C(O)C=C4)C1=O |  |  |
|  |  |  |  | O=C1C(C2=CC=C(O)C=C2)=C(OCO3)C3=C(C4=CC=C(O)C=C4)C1=O |  |  |
| 6461 | 0 | 423.122 | C27H18O5 | / | 5-O-Methylxylerythrin [2] | p-Terphenyls |
|  |  |  |  | O=C1C(C2=CC=C(O)C=C2)=C(C(O1)=C3C4=CC=CC=C4)C(C5=CC=CC=C5)=C(OC)C3=O |  |  |
|  |  |  |  | O=C1C(C2=CC=C(O)C=C2)=C(C(O1)=C3C4=CC=CC=C4)C(C5=CC=CC=C5)=C(OC)C3=O |  |  |
| 6390 | 1 | 437.1012 | C27H16O6 | / | Peniophorinin [2] | p-Terphenyls |
|  |  |  |  | O=C1C(C2=CC=C(O)C=C2)=C(C(O1)=C3C4=CC=CC=C4)C(C5=C6C=C(O)C=C5)=C(CO6)C3=O |  |  |
|  |  |  |  | O=C1C(C2=CC=C(O)C=C2)=C(C(O1)=C3C4=CC=CC=C4)C(C5=C6C=C(O)C=C5)=C(CO6)C3=O |  |  |
| 6372 | 1 | 409.1063 | C26H16O5 | / | Xylerythrin [2] | p-Terphenyls |
|  |  |  |  | O=C1C(C2=CC=C(O)C=C2)=C(C(O1)=C3C4=CC=CC=C4)C(C5=CC=CC=C5)=C(O)C3=O |  |  |
|  |  |  |  | O=C1C(C2=CC=C(O)C=C2)=C(C(O1)=C3C4=CC=CC=C4)C(C5=CC=CC=C5)=C(O)C3=O |  |  |
| 6376 | 1 | 425.1011 | C26H16O6 | / | Peniophorin [2] | p-Terphenyls |
|  |  |  |  | O=C1C(C2=CC=C(O)C=C2)=C(C(O1)=C3C4=CC=CC=C4)C(C5=CC=C(O)C=C5)=C(O)C3=O |  |  |
|  |  |  |  | O=C1C(C2=CC=C(O)C=C2)=C(C(O1)=C3C4=CC=CC=C4)C(C5=CC=C(O)C=C5)=C(O)C3=O |  |  |

## Cluster H


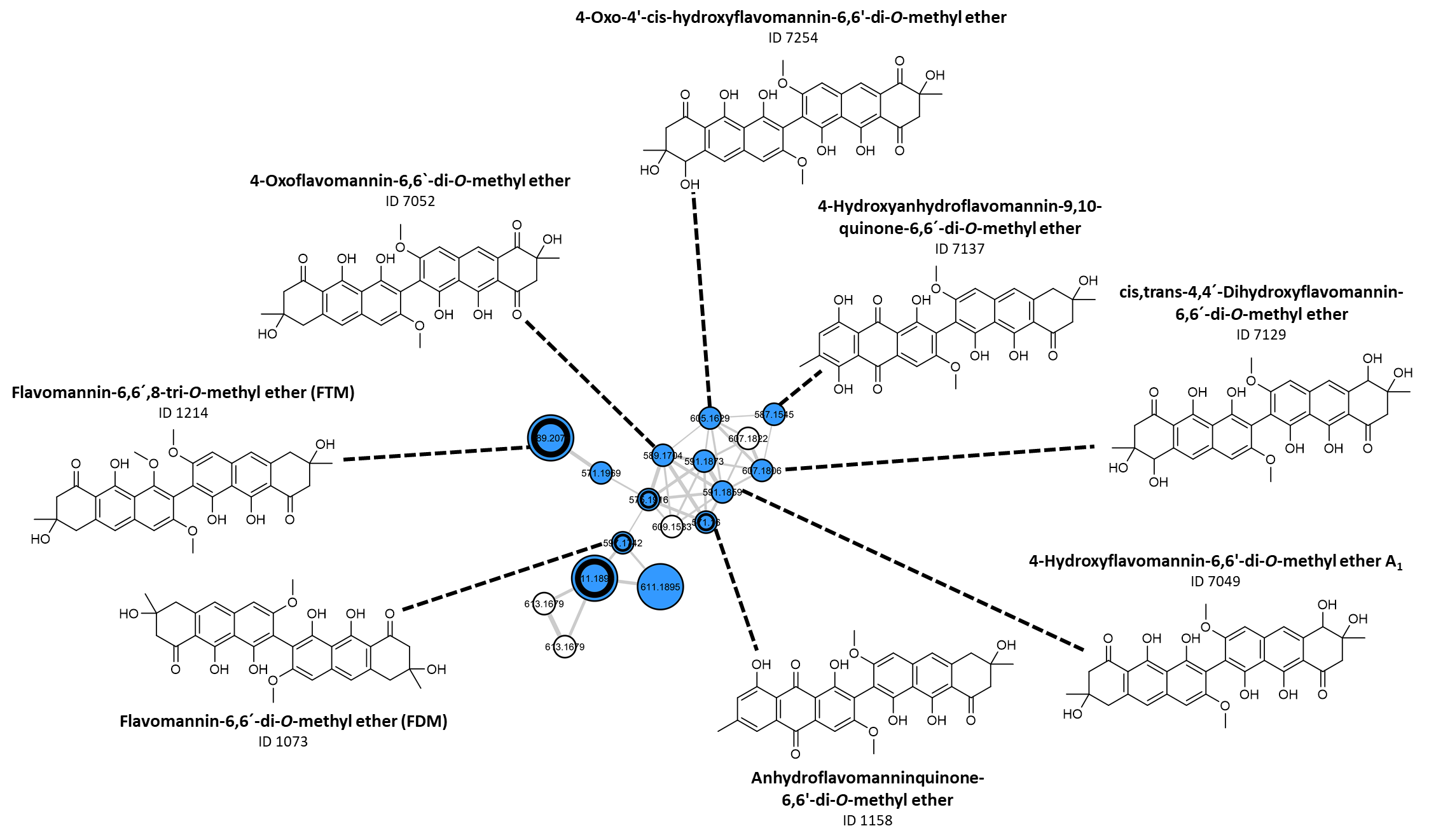


**Figure S7.** Annotated Cluster H. Each node displays the precursor mass (please zoom in). Out of the different molecule annotations listed in the table below (Table S8), the chemical structure was depicted, which was deemed most probable. Illustration code: blue node filling … acetate-malonate pathway, large node … “VIS-Signal” present, small node … “VIS-Signal” absent, black ring … high photocytotoxicity.

**Table S8.** The annotation results for Cluster H presented as the respective compound ID (shared name), the “VIS-Signal”, m/z, the molecular formula, the SMILES (top 🡪 bottom: GNPS, In-house library, ISDB-DNP; Black color … SMILES belonging to the chemical structure drawn in the figure, Grey color … additional hits, Green color … identical output, / … no hit at all), the name, and the NPClassifier “class”.

| **Comp.ID (shared name)** | **VIS** | **m/z** | **Molecular formula** | **Annotation (SMILES): GNPS \| In-house library \| ISDB-DNP** | **Name [Identification level]** | **NPClassifier "class"** |
| --- | --- | --- | --- | --- | --- | --- |
| 1214 | 1 | 611.1898 | C33H32O10 | / | Flavomannin-6,6´,8-tri-*O*-methyl ether [2] | Anthraquinones and anthrones |
|  |  |  |  | O=C1C[C@@](C)(O)C([H])C2=CC3=CC(OC)=C(C(C(OC)=C4)=C(O)C(C4=C5)=C(O)C6=C5C([H])[C@](C)(O)CC6=O)C(OC)=C3C(O)=C21 |  |  |
|  |  |  |  | O=C1C[C@@](C)(O)C([H])C2=CC3=CC(OC)=C(C(C(OC)=C4)=C(O)CC4=C5)=C(O)C6=C5C([H])[C@](C)(O)CC6=O)C(OC)=C3C(O)=C21 |  |  |
| 1158 | 0 | 571.16 | C32H26O10 | / | Anhydroflavomanninquinone-6,6'-di-*O*-methyl ether [2] | Anthraquinones and anthrones |
|  |  |  |  | OC1=C(C2=C(OC)C=C(C(C(C=C(C)C=C3O)=C3C4=O)=O)C4=C2O)C(OC)=CC5=CC6=C(C(O)=C51)C(C[C@](O)(C)C6[H])=O |  |  |
|  |  |  |  | OC1=C(C2=C(OC)C=C(C(C(C=C(C)C=C3O)=C3C4=O)=O)C4=C2O)C(OC)=CC5=CC6=C(C(O)=C51)C(C[C@](O)(C)C6[H])=O |  |  |
| 7052 | 0 | 589.1704 | C32H28O11 | / | 4-Oxoflavomannin-6,6`-di-*O*-methyl ether [2] | Naphthoquinones |
|  |  |  |  | OC1=C(C2=C(OC)C=C(C=C(C([C@@](O)(C)CC3=O)=O)C3=C4O)C4=C2O)C(OC)=CC5=CC6=C(C(O)=C51)C(C[C@](O)(C)C6)=O |  |  |
|  |  |  |  | COC1=CC(=O)c2c(cc3c(c2O)-c2c(OC)cc4cc5c(c(O)c4c2OC(=O)C[C@](C)(O)C3)C(=O)C[C@@](C)(O)C5)C1=O |  |  |
| 7049 | 0 | 591.1859 | C32H30O11 | / | 4-Hydroxyflavomannin-6,6'-di-*O*-methyl ether A1 [2] | Anthraquinones and anthrones |
|  |  |  |  | OC1=C(C2=C(OC)C=C(C=C([C@@H](O)[C@@](O)(C)CC3=O)C3=C4O)C4=C2O)C(OC)=CC5=CC6=C(C(O)=C51)C(C[C@](O)(C)C6)=O |  |  |
|  |  |  |  | OC1=C(C2=C(OC)C=C(C=C([C@@H](O)[C@@](O)(C)CC3=O)C3=C4O)C4=C2O)C(OC)=CC5=CC6=C(C(O)=C51)C(C[C@](O)(C)C6)=O |  |  |
| 7254 | 0 | 605.1629 | C32H28O12 | / | 4-Oxo-4'-cis-hydroxyflavomannin-6,6'-di-*O*-methyl ether [2] | Duclauxin and derivatives |
|  |  |  |  | OC1=C(C2=C(OC)C=C(C=C(C([C@@](O)(C)CC3=O)=O)C3=C4O)C4=C2O)C(OC)=CC5=CC6=C(C(O)=C51)C(C[C@](O)(C)[C@H]6O)=O |  |  |
|  |  |  |  | CO[C@@]12c3c(C)cc(O)c4c3C3=C(O[C@](C)(O)C[C@H]3OC4=O)[C@@H]1[C@]13COC(=O)c4c(O)cc(C)c(c41)C(=O)[C@H]2[C@@H]3OC(C)=O |  |  |
| 7129 | 0 | 607.1806 | C32H30O12 | / | cis,trans-4,4´-Dihydroxyflavomannin-6,6´-di-*O*-methyl ether [2] | nan |
|  |  |  |  | OC1=C(C2=C(OC)C=C(C=C([C@H](O)[C@@](O)(C)CC3=O)C3=C4O)C4=C2O)C(OC)=CC5=CC6=C(C(O)=C51)C(C[C@](O)(C)[C@@H]6O)=O |  |  |
|  |  |  |  | COc1cc(OC)c2c3c(c(O)c(-c4c(OC)cc5cc(O)c6c(c5c4OC)OC(C)(O)CC6=O)c2c1)C(=O)CC(C)(O)O3 |  |  |
| 7137 | 0 | 587.1545 | C32H26O11 | / | 4-Hydroxyanhydroflavomannin-9,10-quinone-6,6´-di-*O*-methyl ether [2] | Anthraquinones and anthrones |
|  |  |  |  | OC1=C(C2=C(OC)C=C(C(C(C(O)=C(C)C=C3O)=C3C4=O)=O)C4=C2O)C(OC)=CC5=CC6=C(C(O)=C51)C(C[C@](O)(C)C6)=O |  |  |
|  |  |  |  | COc1cc(O)c(Oc2cc(C)cc(O)c2C(=O)O)c(C2=C(c3c(O)cc(C)cc3O)c3c(O)cc(OC)cc3C2=O)c1 |  |  |
| 1037 | 0 | 575.1916 | C32H30O10 | / | Flavomannin-6,6´-di-*O*-methyl ether (FDM) [2] | Anthraquinones and anthrones |
|  |  |  |  | OC1=C(C2=C(OC)C=C(C=C(C[C@](C)(O)CC3=O)C3=C4O)C4=C2O)C(OC)=CC5=CC6=C(C(O)=C51)C(C[C@](O)(C)C6)=O |  |  |
|  |  |  |  | COc1cc(O)c2c(O)c3c(c(-c4c(OC)cc5cc6c(c(O)c5c4O)C(=O)CC(C)(O)C6)c2c1)CC(C)(O)CC3=O |  |  |

## Cluster I


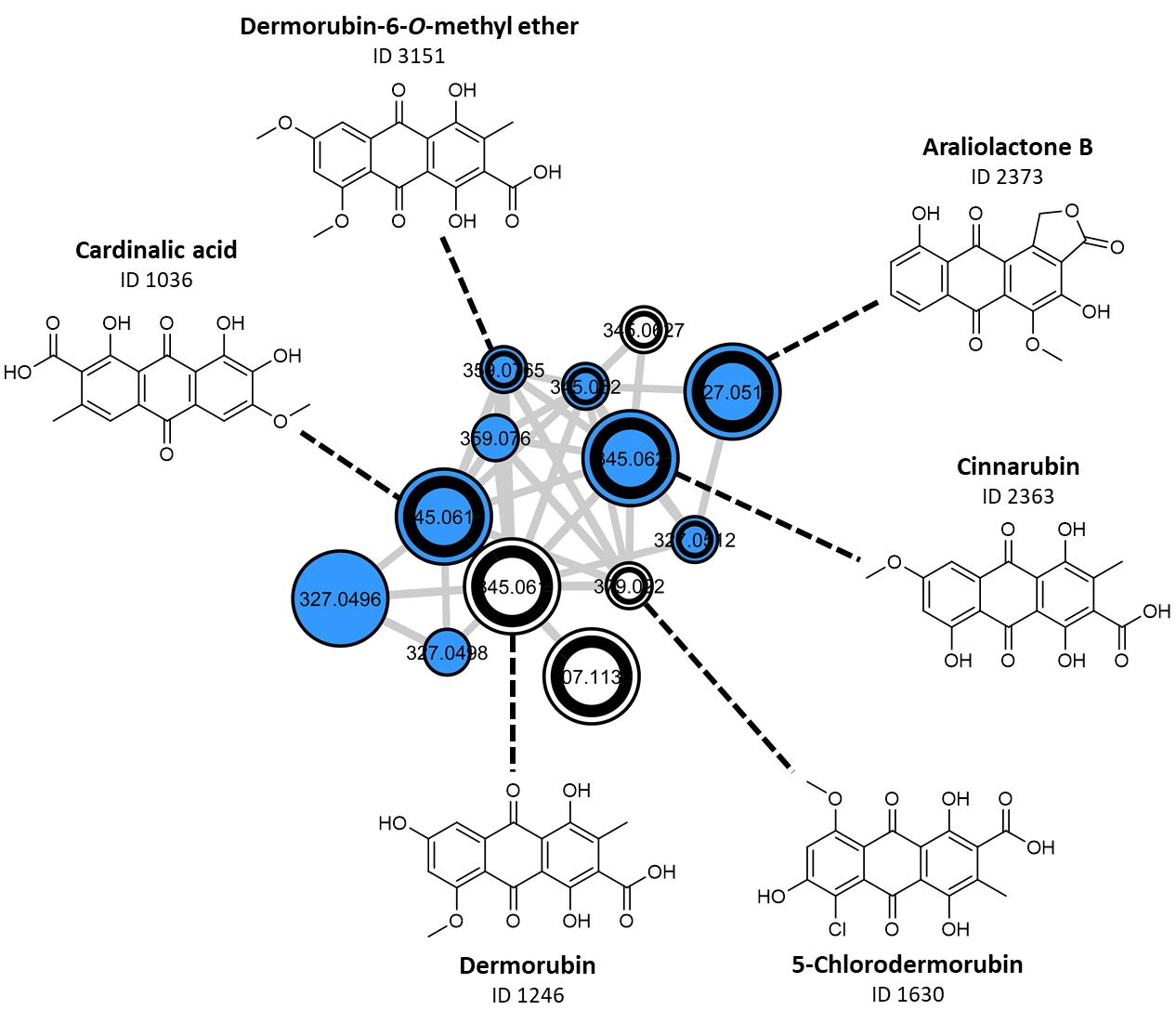


**Figure S8.** Annotated Cluster I. Each node displays the precursor mass (please zoom in). Out of the different molecule annotations listed in the table below (Table S9), the chemical structure was depicted, which was deemed most probable. Illustration code: blue node filling … acetate-malonate pathway, large node … “VIS-Signal” present, small node … “VIS-Signal” absent, black ring … high photocytotoxicity.

**Table S9.** The annotation results for Cluster I presented as the respective compound ID (shared name), the “VIS-Signal”, m/z, the molecular formula, the SMILES (top 🡪 bottom: GNPS, In-house library, ISDB-DNP; Black color … SMILES belonging to the chemical structure drawn in the figure, Grey color … additional hits, Green color … identical output, / … no hit at all), the name, and the NPClassifier “class”.

| **Compound ID (shared name)** | **"VIS-Signal" (yes = 1/no = 0)** | **m/z** | **Molecular formula** | **Annotation (SMILES): GNPS \| In-house library \| ISDB-DNP** | **Name [Identification level]** | **NPClassifier "class"** |
| --- | --- | --- | --- | --- | --- | --- |
| 2373 | 1 | 327.0512 | C17H10O7 | / | Araliolactone B [2] | Anthraquinones and anthrones |
|  |  |  |  | / |  |  |
|  |  |  |  | COc1c(O)c2c(c3c1C(=O)c1cccc(O)c1C3=O)COC2=O |  |  |
| 1036 | 1 | 345.0612 | C17H12O8 | / | Cardinalic acid [2] | Anthraquinones and anthrones |
|  |  |  |  | O=C1C2=C(C=C(C)C(C(O)=O)=C2O)C(C3=CC(OC)=C(O)C(O)=C31)=O |  |  |
|  |  |  |  | O=C1C2=C(C=C(C)C(C(O)=O)=C2O)C(C3=CC(OC)=C(O)C(O)=C31)=O |  |  |
| 2363 | 1 | 345.0627 | C17H12O8 | / | Cinnarubin [2] | Anthraquinones and anthrones |
|  |  |  |  | O=C1C2=C(C(O)=C(C)C(C(O)=O)=C2O)C(C3=CC(OC)=CC(O)=C31)=O |  |  |
|  |  |  |  | COc1cc(O)cc2c1C(=O)c1c(O)c(C(=O)O)c(C)c(O)c1C2=O |  |  |
| 3151 | 0 | 359.0765 | C18H14O8 | / | Dermorubin-6-O-methylether [2] | Flavan-3-ols |
|  |  |  |  | O=C1C2=C(OC)C=C(OC)C=C2C(C3=C1C(O)=C(C(O)=O)C(C)=C3O)=O |  |  |
|  |  |  |  | O=c1oc2cc(O)c3c(c2cc1O)OC(c1ccc(O)c(O)c1)C(O)C3 |  |  |
| 1246 | 1 | 345.061 | C17H12O8 | / | Dermorubin [1, isolated compound] | / |
|  |  |  |  | O=C1C2=C(C(O)=C(C)C(C(O)=O)=C2O)C(C3=CC(O)=CC(OC)=C31)=O |  |  |
|  |  |  |  | / |  |  |
| 1630 | 0 | 379.022 | C17H11ClO8 | / | 5-Chlorodermorubin [2] | / |
|  |  |  |  | O=C1C2=C(C(O)=C(C)C(C(O)=O)=C2O)C(C3=C(Cl)C(O)=CC(OC)=C31)=O |  |  |
|  |  |  |  | / |  |  |

# (Photo)biological evaluation

**Table S10.** The results of the (photo)cytotoxicity assay of the extracts of D. concentrica (PE and MeOH) and B. inquinans (MeOH). The extracts´ dark cytotoxicity (D) as well as their toxicity against the three cancer cell lines A549, AGS, and T24 after irradiation with blue light irradiation (BL, λ = 468 ± 27 nm, 9.3 J/cm²) was evaluated. EC_50_-values including their 95% confidence intervals are given in µg/mL. The ratio of cells killed in the dark versus cells killed upon light-activated treatment (i.e. photoindex (P.I.)) is depicted as well.

| **EC_50_ [µg/mL]** | **A549 (BL, 468 nm)** | | **A549 (D)** | | **P.I.** | **AGS (BL, 468 nm)** | | **AGS (D)** | | **P.I.** | **T24 (BL, 468 nm)** | | **T24 (D)** | | **P.I.** |
| --- | --- | --- | --- | --- | --- | --- | --- | --- | --- | --- | --- | --- | --- | --- | --- |
| ***D. concentrica* (PE)** | 14.0 | 1.3 | 30.8 | 3.2 | 2.2 | 7.3 | 0.9 | 12.2 | 2.4 | 1.7 | 6.0 | 0.5 | 17.9 | 2.7 | 3.0 |
|  |  | 1.2 |  | 2.9 |  |  | 0.8 |  | 2.0 |  |  | 0.4 |  | 2.4 |  |
| ***D. concentrica* (MeOH)** | 41.9 | 11.3 | >55.0 |  | >1.3 | 24.0 | 6.4 | >55.0 |  | >2.3 | 7.9 | 1.8 | >55.0 |  | >7.0 |
|  |  | 8.9 |  |  |  |  | 5.0 |  |  |  |  | 1.5 |  |  |  |
| ***B. inquinans* (MeOH)** | 48.4 | 8.3 | >55.0 |  | >1.1 | 26.8 | 8.1 | >55.0 |  | >2.1 | 19.3 | 3.2 | >55.0 |  | >2.8 |
|  |  | 7.1 |  |  |  |  | 6.2 |  |  |  |  | 2.7 |  |  |  |

# Micrographs (48 hours after irradiation) – *B. inquinans*, *D. concentrica*


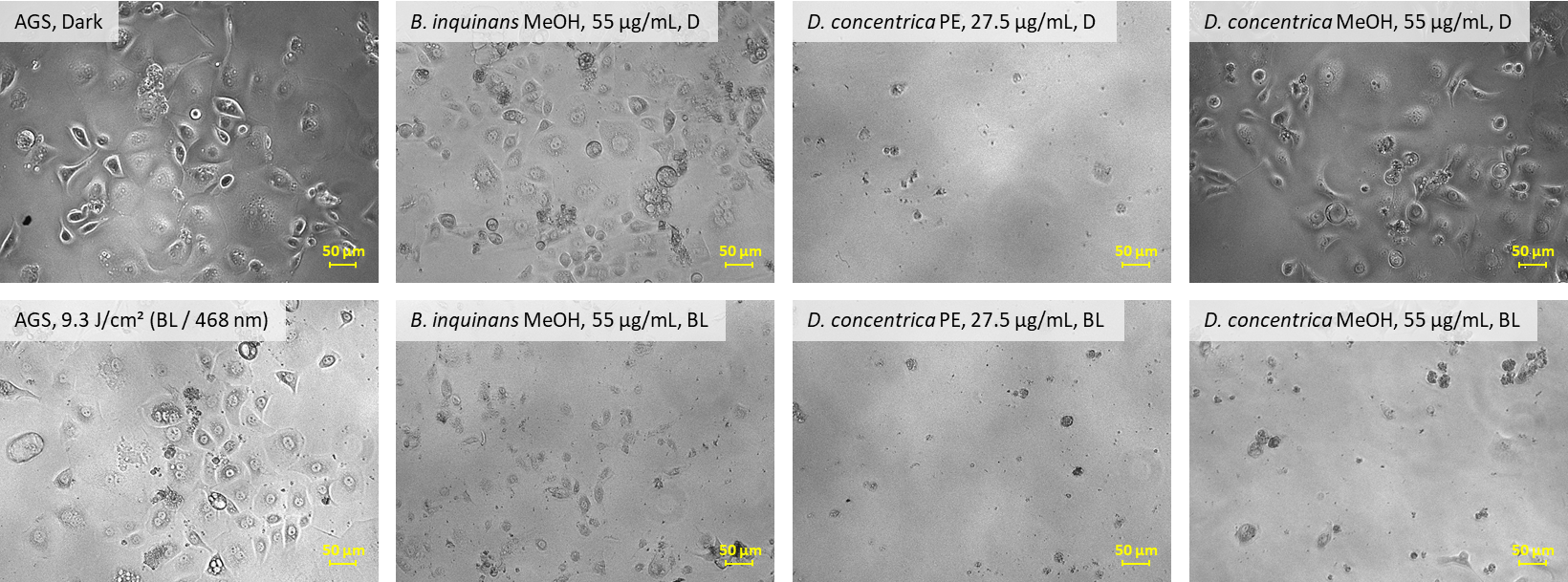


**Figure S9.** Micrographs of cells of the stomach cancer cell line (AGS / human Caucasian gastric adenocarcinoma, 200x magnification) treated (24 h) with the extracts of B. inquinans (MeOH: 55 µg/mL) and D. concentrica (PE: 27.5 µg/mL, MeOH: 55 µg/mL). The upper line of pictures shows treated cells in the dark, the lower after irradiation with blue light (468 nm, 9.3 J/cm²).


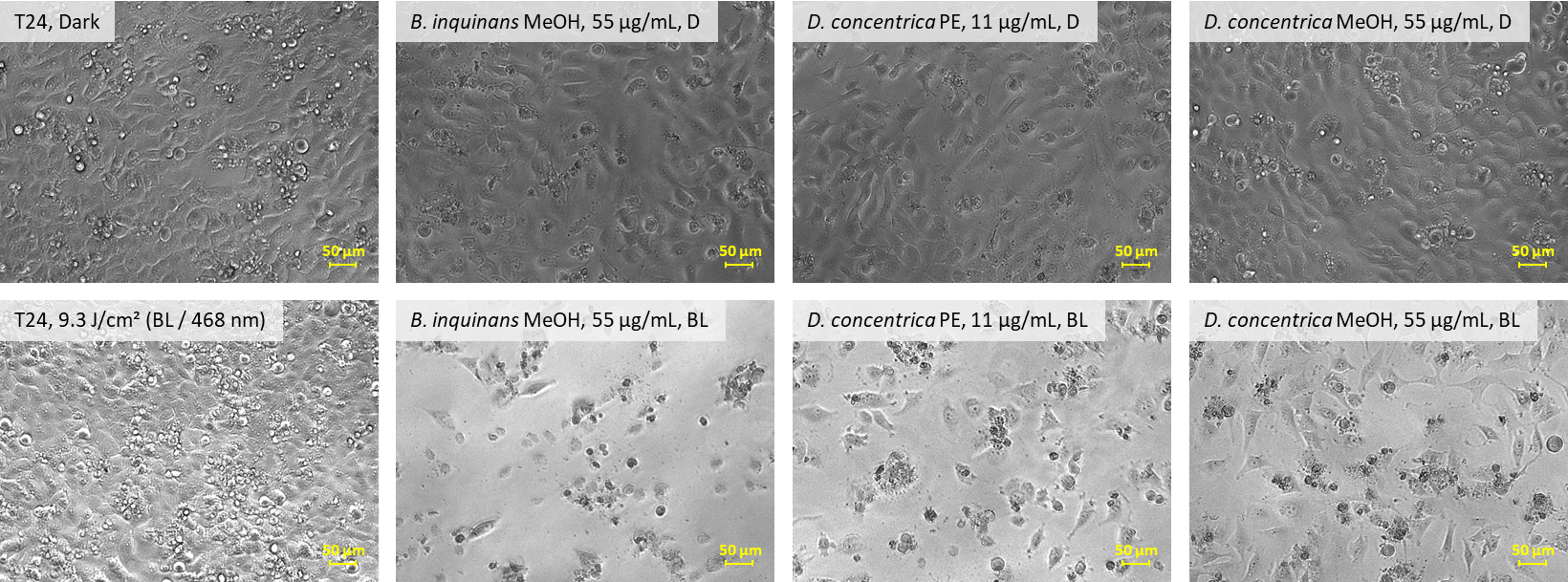


**Figure S10.** Micrographs of cells of the bladder cancer cell line (T24 / human bladder carcinoma, 200x magnification) treated (24 h) with the extracts of B. inquinans (MeOH: 55 µg/mL) and D. concentrica (PE: 11 µg/mL, MeOH: 55 µg/mL). The upper line of pictures shows treated cells in the dark, the lower after irradiation with blue light (468 nm, 9.3 J/cm²).


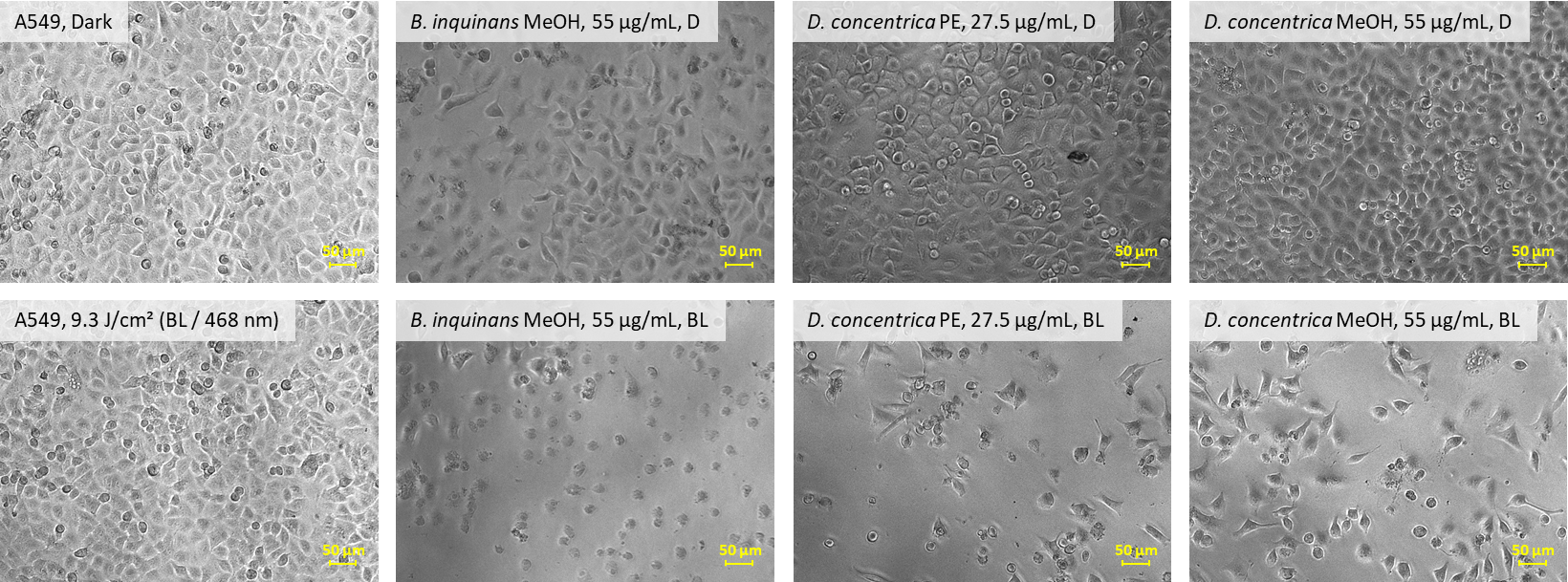


**Figure S11.** Micrographs of cells of the lung cancer cell line (A549 / human Caucasian lung carcinoma, 200x magnification) treated (24 h) with the extracts of B. inquinans (MeOH: 55 µg/mL) and D. concentrica (PE: 27.5 µg/mL, MeOH: 55 µg/mL). The upper line of pictures shows treated cells in the dark, the lower after irradiation with blue light (468 nm, 9.3 J/cm²).
